# Diets for weight management in adults with type 2 diabetes: an umbrella review of published meta-analyses and systematic review of trials of diets for diabetes remission

**DOI:** 10.1007/s00125-021-05577-2

**Published:** 2021-11-17

**Authors:** Chaitong Churuangsuk, Julien Hall, Andrew Reynolds, Simon J. Griffin, Emilie Combet, Michael E. J. Lean

**Affiliations:** 1 Human Nutrition, School of Medicine, Dentistry and Nursing, College of Medical, Veterinary and Life Sciences, University of Glasgow, Glasgow, UK; 2 Division of Internal Medicine, Faculty of Medicine, Prince of Songkla University, Hat Yai, Thailand; 3 Department of Medicine, University of Otago, Dunedin, Otago New Zealand; 4 Edgar National Centre for Diabetes and Obesity Research, University of Otago, Dunedin, Otago New Zealand; 5 MRC Epidemiology Unit, Institute of Metabolic Science, School of Clinical Medicine, University of Cambridge, Cambridge, UK; 6 Primary Care Unit, Department of Public Health and Primary Care, School of Clinical Medicine, University of Cambridge, Cambridge, UK

**Keywords:** Diet, Evidence-based guidelines, Informed clinical practice, Meta-analysis, Quality assessment, Randomised trial, Remission, Systematic review, Type 2 diabetes, Weight loss

## Abstract

**Aims/hypothesis:**

Weight reduction is fundamental for type 2 diabetes management and remission, but uncertainty exists over which diet type is best to achieve and maintain weight loss. We evaluated dietary approaches for weight loss, and remission, in people with type 2 diabetes to inform practice and clinical guidelines.

**Methods:**

First, we conducted a systematic review of published meta-analyses of RCTs of weight-loss diets. We searched MEDLINE (Ovid), PubMed, Web of Science and Cochrane Database of Systematic Reviews, up to 7 May 2021. We synthesised weight loss findings stratified by diet types and assessed meta-analyses quality with A Measurement Tool to Assess Systematic Reviews (AMSTAR) 2. We assessed certainty of pooled results of each meta-analysis using Grading of Recommendations, Assessment, Development and Evaluations (GRADE) (PROSPERO CRD42020169258). Second, we conducted a systematic review of any intervention studies reporting type 2 diabetes remission with weight-loss diets, in MEDLINE (via PubMed), Embase and Cochrane Central Register of Controlled Trials, up to 10 May 2021. Findings were synthesised by diet type and study quality (Cochrane Risk of Bias tool 2.0 and Risk Of Bias In Non-randomised Studies – of Interventions [ROBINS-I]), with GRADE applied (PROSPERO CRD42020208878).

**Results:**

We identified 19 meta-analyses of weight-loss diets, involving 2–23 primary trials (*n* = 100–1587), published 2013–2021. Twelve were ‘critically low’ or ‘low’ AMSTAR 2 quality, with seven ‘high’ quality. Greatest weight loss was reported with very low energy diets, 1.7–2.1 MJ/day (400–500 kcal) for 8–12 weeks (high-quality meta-analysis, GRADE low), achieving 6.6 kg (95% CI −9.5, −3.7) greater weight loss than low-energy diets (4.2–6.3 MJ/day [1000–1500 kcal]). Formula meal replacements (high quality, GRADE moderate) achieved 2.4 kg (95% CI −3.3, −1.4) greater weight loss over 12–52 weeks. Low-carbohydrate diets were no better for weight loss than higher-carbohydrate/low-fat diets (high quality, GRADE high). High-protein, Mediterranean, high-monounsaturated-fatty-acid, vegetarian and low-glycaemic-index diets all achieved minimal (0.3–2 kg) or no difference from control diets (low to critically low quality, GRADE very low/moderate). For type 2 diabetes remission, of 373 records, 16 met inclusion criteria. Remissions at 1 year were reported for a median 54% of participants in RCTs including initial low-energy total diet replacement (low-risk-of-bias study, GRADE high), and 11% and 15% for meal replacements and Mediterranean diets, respectively (some concerns for risk of bias in studies, GRADE moderate/low). For ketogenic/very low-carbohydrate and very low-energy food-based diets, the evidence for remission (20% and 22%, respectively) has serious and critical risk of bias, and GRADE certainty is very low.

**Conclusions/interpretation:**

Published meta-analyses of hypocaloric diets for weight management in people with type 2 diabetes do not support any particular macronutrient profile or style over others. Very low energy diets and formula meal replacement appear the most effective approaches, generally providing less energy than self-administered food-based diets. Programmes including a hypocaloric formula ‘total diet replacement’ induction phase were most effective for type 2 diabetes remission. Most of the evidence is restricted to 1 year or less. Well-conducted research is needed to assess longer-term impacts on weight, glycaemic control, clinical outcomes and diabetes complications.

**Graphical abstract:**

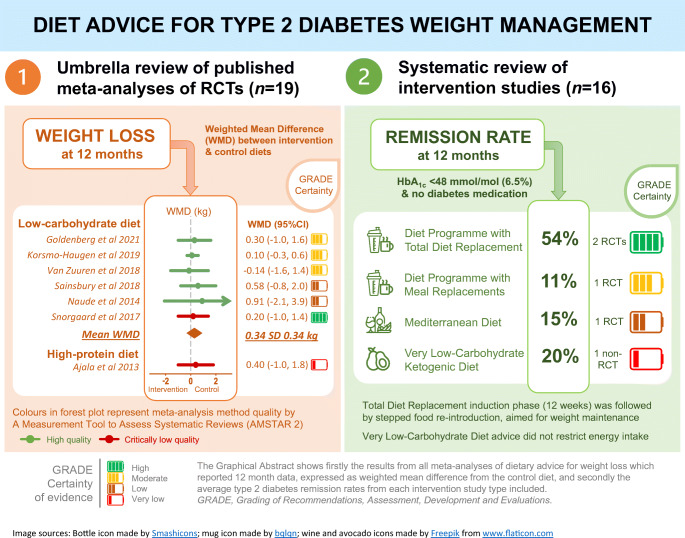

**Supplementary Information:**

The online version contains peer-reviewed but unedited supplementary material available at 10.1007/s00125-021-05577-2.

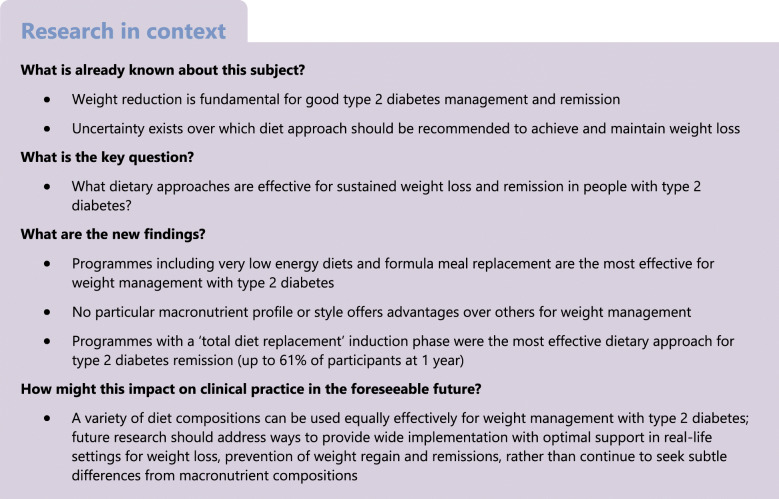



## Introduction

Type 2 diabetes has both environmental and genetic contributors, the global epidemic consistently following obesity. Its onset is primarily driven by weight gain to an excessive level for that individual, in a complex disease process involving gut hormones, low-grade inflammation and metabolites, possibly including some from the gut microbiota [1]. Ectopic fat accumulation in liver, pancreas and muscle impairs organ functions, resulting in hyperglycaemia, commonly associated with hypertension and dyslipidaemia [2, 3]. Type 2 diabetes requires lifelong management, but disabling and life-shortening complications occur despite treatment [4]. Without strategic commitment, internationally, to effective preventive actions, type 2 diabetes will affect 629 million people worldwide by 2045 [5].

Weight loss improves all weight-related risk factors and reduces medication load. During an intensive weight loss programme, or early after bariatric surgery, there are already significant improvements in hepatic and muscle insulin sensitivity, and pancreatic first-phase insulin secretion, with rapid loss of ectopic fat from skeletal muscle and liver [2, 3, 6]. A non-diabetic state can be restored for 2 years for 70–80% of people with type 2 diabetes by interventions that maintain over 10 kg weight loss (36/149, 24% of participants in the Diabetes Remission Clinical Trial [DiRECT]) [7], which sustains loss of ectopic fat, reversing the pathophysiology and normalising pancreas morphology [8].

Awareness of the benefits of weight loss for type 2 diabetes is high, but both patients and healthcare practitioners currently lack authoritative guidance over diets [9]. Current guidelines state that various dietary strategies may be effective, and stress personalising weight management, to take account of social situations, but do not provide guidance over diet compositions [9, 10]. Consequently, practice can be led by distorted evidence and claims.

Adhering to any energy-reduced diet will inevitably generate and sustain weight loss, whether defined by restriction of energy, of food groups or of specific nutrients, provided that there is incomplete compensation in energy intake and expenditure. In practice, adherence and weight losses vary widely within the same programme, and comparisons between diets often appear to have conflicting results [11]. Metabolic diversity in response to specific nutrient contents has been postulated, but possibly overwhelmed or confounded by mixed behavioural responses to dietary advice. Unless carefully designed, some diets may achieve negative energy imbalance but lack essential micronutrients [12, 13] or introduce adverse health effects through other pathways [14–16]. Furthermore, short-term results may not be sustained, potentially requiring additional behavioural approaches for long-term maintenance. While different strategies may work better for some individuals (or some practitioners) than others, there may be preferred diet compositions to optimise weight control [17].

Guideline development has been difficult because systematic reviews and meta-analyses of diet types, themselves open to bias, have appeared conflicting [11]. To resolve these uncertainties and to inform clinical decision making and guideline development as part of a programme of work to update the EASD dietary recommendations, we conducted an umbrella review, to collate and critically appraise all available systematic reviews with meta-analyses of dietary interventions for weight loss in people with type 2 diabetes. As remission of diabetes is now an important goal for weight management, we also conducted a new systematic review and quality appraisal of published intervention studies of non-surgical dietary approaches for type 2 diabetes remission.

## Methods

### Protocol and registration

This paper focuses on dietary strategies for weight loss and type 2 diabetes remission and includes two systematic reviews: (1) a systematic ‘umbrella review’ of published meta-analyses of RCTs of diets for weight loss in people with type 2 diabetes (PROSPERO CRD42020169258); (2) a systematic review of any intervention studies which report type 2 diabetes remission (PROSPERO CRD42020208878). Our paper is written in accordance with the Preferred Reporting Items for Systematic Reviews and Meta-Analyses (PRISMA) 2020 [18] and the Synthesis Without Meta-analysis in systematic reviews: reporting guideline [19].

**Fig. 1:**
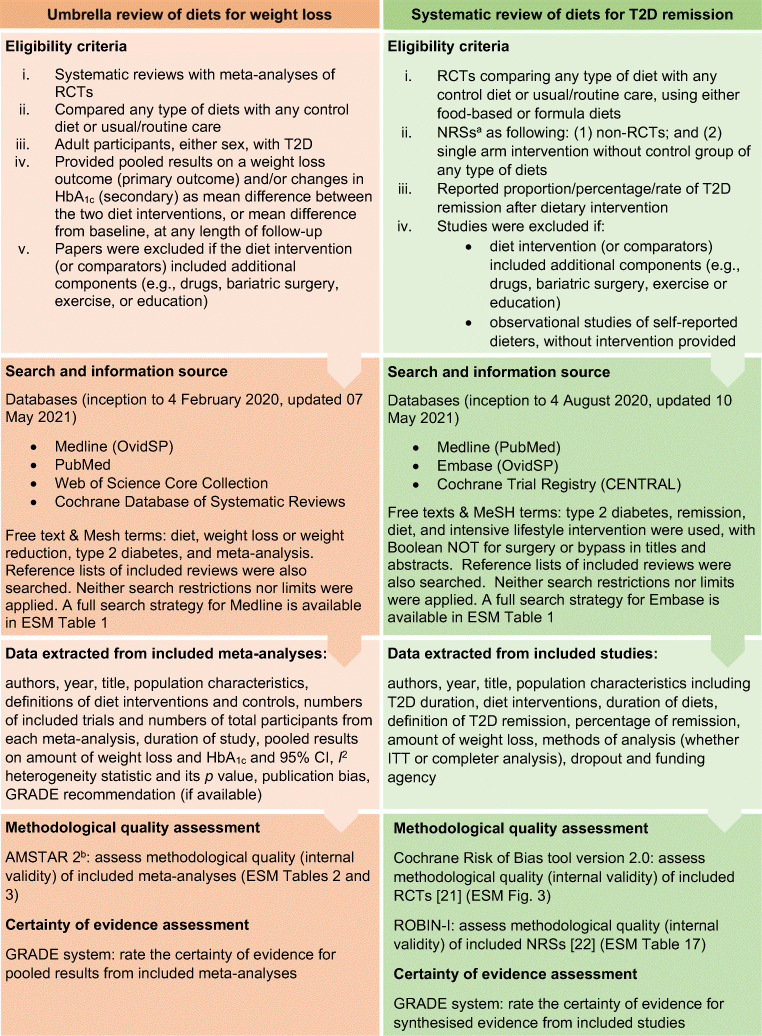
Summary of the methodological processes of both systematic reviews. Detailed methods are presented in the ESM Methods. ^a^These types of NRSs provided intervention to participants and assessed outcomes at designated specific time points (baseline and at the end of intervention), although they could suffer from selection bias and confounding bias. ^b^AMSTAR 2 level of quality assessment: high quality—the meta-analysis provides an accurate and comprehensive summary of the results of the available studies that addresses the question of interest; moderate—the meta-analysis has more than one weakness, but no critical flaws. It may provide an accurate summary of the results of the available studies; low—the meta-analysis has a critical flaw and may not provide an accurate and comprehensive summary of the available studies that address the question of interest; or critically low—the meta-analysis has more than one critical flaw and should not be relied on to provide an accurate and comprehensive summary of the available studies. CENTRAL, Cochrane Central Register of Controlled Trials; ROBINS-I, Risk Of Bias In Non-randomised Studies – of Interventions; T2D, type 2 diabetes

Detailed methods of both systematic reviews are presented in the electronic supplementary material (ESM) Methods and summarised in Fig. 1. The search strategy is in ESM Table 1.

### (1) Umbrella review of published meta-analyses

We searched MEDLINE (Ovid), PubMed, Web of Science and Cochrane Database of Systematic Reviews, up to 7 May 2021, for eligible meta-analyses of RCTs of dietary advice for weight loss.

#### Data synthesis

Synthesised findings (weight loss and HbA_1c_) from each meta-analysis included are grouped by diet type, ranked by overall methodological quality using A Measurement Tool to Assess Systematic Reviews (AMSTAR) 2 (ESM Tables 2, 3) and categorised into four levels: high, moderate, low and critically low. Grading of Recommendations, Assessment, Development and Evaluations (GRADE) evaluates the certainty of evidence of pooled results (ESM Table 4).

Planned analysis of associations between changes in energy intake and weight changes from baseline, to differentiate effects of energy restriction and dietary regimen, proved impossible from the published information.

### (2) Systematic review of diets for type 2 diabetes remission

We searched MEDLINE (via PubMed), Embase and Cochrane Central Register of Controlled Trials, up to 10 May 2021, for any intervention studies reporting type 2 diabetes remission with weight loss dietary advice. We first included RCTs reporting type 2 diabetes remission as the primary outcome, the design most likely to provide trustworthy evidence. However, as few such RCTs have been conducted, we also evaluated non-randomised studies (NRSs) to capture the totality of the evidence for ‘best available advice’ to inform practice and policy [20]. Cochrane Risk of Bias tool 2.0 [21] and Risk Of Bias In Non-randomised Studies – of Interventions (ROBINS-I) [22] were used for quality assessment of RCTs and NRSs, respectively.

#### Data synthesis

Remission of diabetes was reported as percentage from intention to treat (ITT), including all participants. If only completers were reported, we computed an ITT figure assuming participants lost to follow-up all failed to achieve remission (as in the published RCTs). We summarised effect estimates (e.g., median and interquartile ranges), without performing meta-analysis, due to the limited number and heterogeneity of studies [23]. GRADE assesses the certainty of synthesised findings [24].

For the main synthesis, priority was set to RCTs reporting 1 year outcome and low risk of bias. If there was no RCT for a particular diet, synthesis findings were drawn from NRSs with low, followed by high, risk of bias. If both RCTs and NRSs were available for a diet, RCTs were used for synthesised findings and NRSs as supportive evidence [20]. Heterogeneity was explored according to hypothesised effect modifiers: study design, duration of type 2 diabetes and ethnicity.

## Results

### (1) Umbrella review of published meta-analyses of RCTs of diets for weight loss and glycaemic control

#### Identification of meta-analyses

We retrieved 1064 records, including all languages. After removing duplicates, we screened 690 titles and abstracts, and assessed 59 full texts for eligibility. Excluded full texts, with reasons, are shown in ESM Table 5. We included a total of 21 systematic reviews (with 19 meta-analyses) for data synthesis and quality assessment (ESM Fig. 1).

#### Characteristics of included meta-analyses

Of the 19 meta-analyses (Table 1, ESM Table 6), 18 reported direct comparisons of specific diets. Control diets varied, either usual/routine care or a particular dietary regimen. One meta-analysis used a network method to consider both direct and indirect comparisons between multiple diets (Mediterranean diets, low-carbohydrate diets [LCDs], low-fat diets [LFDs], high-carbohydrate diets and usual diets) [25]. Most meta-analyses were of critically low (*n* = 7; [25–31]) to low quality (*n* = 5; [32–36]). Only seven meta-analyses (LCDs, *n* = 5 [37–40, 41]; liquid meal replacement, *n* = 1 [42]; very low energy diet (VLED), *n* = 1 [43]) were assessed as high quality. The ESM Results and ESM Tables 7–10 present methodological quality, heterogeneity and overlaps in source trials of meta-analyses included in the umbrella review.

**Table 1:** Characteristics of included meta-analyses of RCTs of dietary weight management in type 2 diabetes

Authors, yr	AMSTAR 2 quality	Protocol and no. of DBs/registries searched^a^	No. of RCTs (*N* individuals) for weight loss outcome^b^	Publication bias	INT diets (criteria)	INT: reported macronutrient intake	CON diets (criteria)	CON diet: reported macronutrient intake	Criteria for duration	Criteria for E restriction	Reported E intake in included RCTs
LCDs											
Goldenberg et al., 2021 [41]	High	Protocol: yes 6 DBs: CENTRAL, MEDLINE, Embase, CINAHL, CAB and grey literature	18 (882) Used data from complete cases, not ITT	Publication bias for weight loss at 6 mo	LCD (<26% E CHO)	<20 to <130 g CHO	≥26% E CHO	NR	>12 wk	NR	NR; included RCTs with either E restriction or ad libitum E intake
Korsmo-Haugen et al., 2019 [37]	High	Protocol: yes 6 DBs: MEDLINE, Embase, CENTRAL, CINAHL, Food Science Source and SweMed+	17 (1587)	No publication bias	LCD (<40% E CHO)	5–40% E CHO 15–30% protein 30–50% fat	>40% E CHO	45–60% CHO 10–20% protein 20–36% fat	>3 mo	NR	NR; included RCTs with either E restriction or ad libitum E intake
van Zuuren et al., 2018 [38]	High	Protocol: yes 11 DBs^c^ 5 trial registries	16 (1000)	<10 studies included, did not conduct test for publication bias	LCD (<40% E CHO)	NR	LFD (<30% E)	NR	≥4 wk	NR	NR; included RCTs with either E restriction or ad libitum E intake
Sainsbury et al., 2018 [39]	High	Protocol: yes 5 DBs: MEDLINE, Embase, CINAHL, Global Health and CENTRAL	7 (521)^d^ for low- and very low-CHO diets by their definition	Publication bias for HbA_1c_ at 3 mo No publication bias for HbA_1c_ at 6 or 12 mo Did not assess for weight loss	(1) Very low CHO (<50 g CHO) (2) LCD (<130 g CHO)	14–20% E CHO 20–120 g CHO 28–30% protein 35–58% fat	High-CHO diet (>45% E)	45–55% CHO 10–20% protein <30% fat	>3 mo	NR	INT: E intake was mostly ad libitum CON: E restriction: 6.3–7.5 MJ/d (1500–1800 kcal/d) or 2.1 MJ (500 kcal) deficit
Naude et al., 2014 [40]	High	Protocol: yes 3 DBs: MEDLINE, Embase and CENTRAL	5 (599)	<10 studies included, did not conduct test for publication bias	LCD (<40% E CHO)	20–40% CHO 30% protein 30–50% fat	High-CHO diet Isoenergetic to INT 45–65% CHO 25–35% fat 10–20% protein	55–60% CHO 30% fat 10–15% protein	>3 mo	NR	INT: 5.3–8.6 MJ (1260–2054 kcal) CON: 5.9–7.5 MJ (1416–1800 kcal)
McArdle et al., 2019 [34]	Low	Protocol: yes 5 DBs: MEDLINE, Embase, CINAHL, Cochrane Library and DARE	13 (706)^d^ for low- and very low-CHO diets by their definition	Did not conduct	(1) Very low CHO (<50 g CHO) (2) LCD (<130 g CHO)	8 RCTs <50 g CHO 4 RCTs 70–130 g CHO 1 RCT unclear amount of CHO	Low-fat, high-CHO, low-GI, high-protein, Mediterranean and ‘healthy eating’	CHO range: 138–232 g (50–60% E) Did not report other macronutrients	>12 wk	NR	NR
Meng et al., 2017 [33]	Low	Protocol: NR 3 DBs: MEDLINE, Embase and the Cochrane Library	8 (590)	No publication bias for weight loss and HbA_1c_	LCD (<130 g or 26% E CHO)	5–20% E CHO <20–130 g CHO	High-CHO diet	45–60% E CHO	NR	NR	NR
Snorgaard et al., 2017 [31]	Critically low	Protocol: NR 3 DBs: Embase, MEDLINE and the Cochrane Library	10 (1376)	Did not conduct	LCD (<45% E CHO)	14–45% CHO 15–28% protein 33–58% fat	High-CHO (45–50% E CHO)	41–55% CHO 15–21% protein 29–37% fat	NR	NR	NR
Fan et al., 2016 [27]	Critically low	Protocol: NR 4 DBs: Embase, PubMed, MEDLINE and Cochrane Library	9 (997)	Stated that publication bias was evaluated but did not report result	LCD (<130 g CHO)	20-–50% CHO or 20–130 g CHO	LFD, high-CHO, ADA diet^e^	50–60% CHO 15–20% protein 25–30% fat	NR	NR	Included RCTs with either E restriction or ad libitum E intake E-restricted trials: INT: 6.3–7.5 MJ/d (1500–1800 kcal); CON: 5.9–7.5 MJ/d (1400–1800 kcal)
High-protein diets											
Pfeiffer et al., 2020 [28]	Critically low	Protocol: NR 1 DB: PubMed	5 (265)	Did not conduct	High-protein diet (>20% E protein), in exchange for CHO	35–45% CHO 25–35% protein 30–35% fat	Lower protein intake (<20% E)	55% CHO 30% fat 15% protein	≥8 wk	NR	INT: 5.1–8.5 MJ/d (1219–2029 kcal) CON: 5.2–7.5 MJ/d (1235–1785 kcal) Included RCTs were of E restriction
Zhao et al., 2018 [30]	Critically low	Protocol: NR 2 DBs: PubMed and Embase	16 (1059)	No publication bias for weight loss, did not assess for HbA_1c_	High-protein diet	30–51% CHO 25–32% protein 18–59% fat	Not specified	40–60% CHO 10–20% protein 10–42% fat	>4 wk	NR	NR
Low-GI diets											
Zafar et al., 2019 [36]	Critically low	Protocol: yes 3 DBs: PubMed, Cochrane Library and Embase 3 trial registries	24 (1488)	No publication bias	Low-GI diet	NR	High-GI, LFD, LCD, low-E weight-loss diets	NR	≥1 wk	NR	NR
Mediterranean diets											
Huo et al., 2015 [32]	Low	Protocol: NR 3 DBs: PubMed, Cochrane Library and Embase	6 (835)	Publication bias for HbA_1c_	Mediterranean-style diets: high vegetable, nuts, legume, fish and fruit intakes, and low red meat intake	NR	Usual diet, usual care, ADA diet^e^, LFD, LCD	NR	>4 wk	NR	NR
Liquid meal replacement											
Noronha et al., 2019 [42]	High	Protocol: yes 3 DBs: MEDLINE, Embase and CENTRAL	9 (931)	<10 studies included, did not conduct test for publication bias	Liquid meal replacement that replaced 1/3 of main meals	Liquid meal represented 20% of total daily E intake (range: 13–47%) 46–52% CHO 20–35% protein 18–33% fat	Low-E weight-loss diets Total E is isoenergetic to INT diet	Total daily E intake 6.3 MJ (1500 kcal) 45–60% CHO 8–31% protein 15–37% fat	>2 wk	NR	Mean 6.3 MJ (1500 kcal) (5.0–6.9 MJ [1195–1659 kcal]) in both arms
VLEDs											
Rehackova et al., 2016 [43]	Low	Protocol: yes 11 DBs^f^	2 (100)	Did not conduct	VLED (<3.3 MJ/d [800 kcal])	NR	Low-E diet (4.2–6.3 MJ/d [1000–1500 kcal])	NR	NR	VLEDs (<3.3 MJ/d [800 kcal])	INT: 1.7–2.1 MJ/d (400–500 kcal) CON: 4.2–6.3 MJ/d (1000–1500 kcal)
High-monounsaturated-fat diets											
Qian et al., 2016 [29]	Critically low	Protocol: NR 3 DBs: PubMed, MEDLINE and CENTRAL	16 (1081)	No publication bias	High-MUFA diet No specified criteria	39% (range: 9.5–45%) CHO 17% (range: 10–41%) protein 43% (range: 30–70%) fat 25% (range: 10–49%) MUFA	High-CHO diet No specified criteria	54% (range: 41–70%) CHO 17% (range: 10–23%) protein 28% (range: 10–39%) fat 11% (range: 1–20%) MUFA	>2 wk	NR	NR
Vegetarian/vegan diets											
Viguiliouk et al., 2019 [35]	Critically low	Protocol: yes 3 DBs: MEDLINE, Embase and CNETRAL	6 (532)	<10 studies included, did not conduct test for publication bias	Vegetarian diet pattern, including vegan to lacto-ovo-vegetarian	60% (range: 49–78%) CHO 15% (range: 12–17%) protein 25% (range: 10–34%) fat 5% (range: 2–9%) SFA 28 g/d (range: 13–39 g/d) fibre	LFD, usual diet	50% (range: 41–65%) CHO 19% (range: 16–22%) protein 30% (range: 19–37%) fat 9% (range: 4–12%) SFA 20 g/d (range: 8–39 g/d) fibre	≥3 wk	NR	NR 8 RCTs E restricted 1 RCT E balanced
Meta-analyses with multiple diets											
Ajala et al., 2013 [26]	Critically low	Protocol: NR 3 DBs: PubMed, Embase and Google Scholar	20 (3073)	Did not conduct	LCD	NR	LFD, low-GI, Mediterranean, high-CHO	NR	≥6 mo	NR	NR
					Low-GI		High-fibre, high-GI, ADA diets^e^				
					Mediterranean		Usual care, ADA diets^e^				
					High-protein diet		Low-protein, high-CHO diets				
Pan et al., 2019 [25]^g^	Critically low	Protocol: yes 3 DBs: PubMed, Embase and CENTRAL	10 (921)	Did not conduct	Mediterranean	NR	High-CHO diet (>55% CHO)	NR	NR	NR	NR
					Mediterranean		LCD				
					Mediterranean		LFD (<30% E)				
					LCD (<26% E /<130 g)		High-CHO diet				
					LFD (<30% E)		LCD (<26% E or <130 g)				
					LFD (<30% E)		High-CHO diet				

^a^See ESM Table 5 for detailed data sources and search used in meta-analyses in the umbrella review

^b^These numbers of RCTs are not all the same as are reported in the original meta-analyses

^c^Eleven databases: MEDLINE, PubMed, Embase, Web of Science, Cochrane Library, CENTRAL, Emcare, Academic Search Premier, ScienceDirect, Latin American and Caribbean Health Science Information database, and Índice Bibliográfico Español en Ciencias de Salud

^d^This meta-analysis also included ‘moderate’-carbohydrate RCTs (26–45% E) and these RCTs were also featured in other meta-analyses as an LCD

^e^Diet according to the recommendation of the ADA [66]

^f^Eleven databases: all EBM Reviews (1991), CAB Abstracts (1973), CINAHL (1994), Embase (1980), HMIC (1979), Ovid MEDLINE(R) In-Process & Other Non-Indexed Citations and Ovid MEDLINE(R) (1946), and PsychINFO (1806). We hand-searched PubMed (1984), Web of Knowledge (1983), The Cochrane library and The Centre for Reviews and Dissemination (CRD)

^g^Network meta-analysis

CENTRAL, Cochrane Central Register of Controlled Trials; CHO, carbohydrate; CON, control; d, day; DB, database; E, energy; GI, glycaemic index; INT, intervention; mo, month; NR, not reported; SFA, saturated fatty acids; wk, week; yr, year

#### Dietary advice for weight loss

**Fig. 2:**
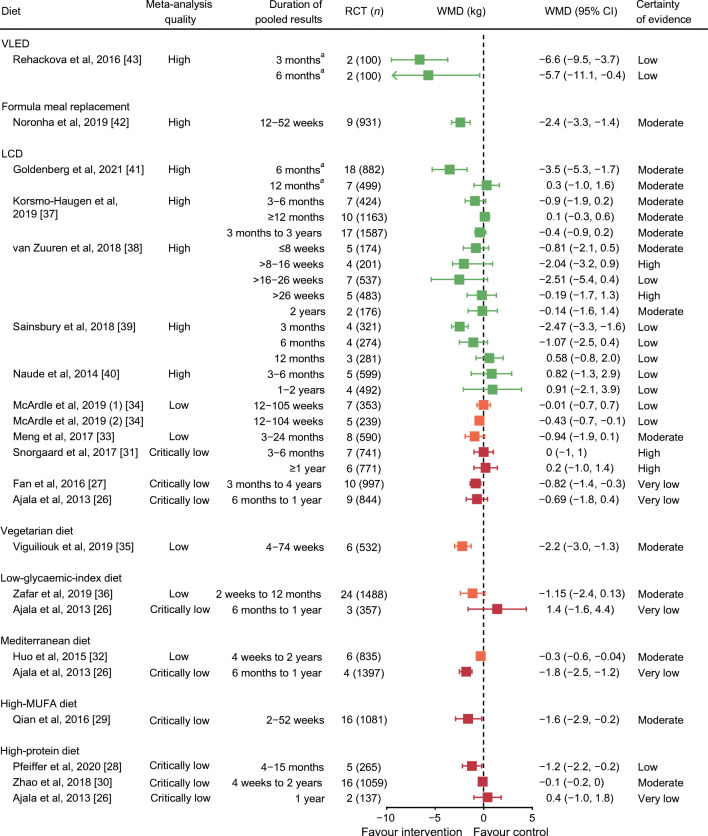
All published meta-analyses of intervention diets vs control diets on weight loss (kg) stratified by overall quality in each diet type using AMSTAR 2 quality (green, high quality; orange, low quality; red, critically low quality). WMDs are presented alongside 95% CIs (error bars). Pooled results of McArdle et al., 2019 [34], Fan et al., 2016 [27], Zafar et al., 2019 [36] and Zhao et al., 2018 [30] are standardised mean differences. ^a^Complete case data. GRADE level for certainty of evidence is rated as follows: ‘high’ indicates that we are very confident that the true effect lies close to that of the estimate of the effect; ‘moderate’ indicates that we are moderately confident in the effect estimate (the true effect is likely to be close to the estimate of the effect, but there is a possibility that it is substantially different); ‘low’ indicates that our confidence in the effect estimate is limited (the true effect may be substantially different from the estimate of the effect); and ‘very low’ indicates that we have very little confidence in the effect estimate (the true effect is likely to be substantially different from the estimate of effect)

**Fig. 3:**
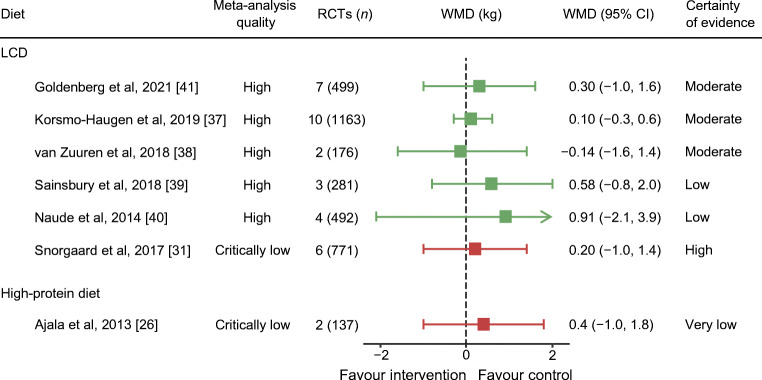
Meta-analyses with source RCTs of 12 months or longer on weight loss (kg) outcome. WMDs are presented alongside 95% CIs (error bars). Different colours indicate meta-analysis quality: green, high quality; red, critically low quality. GRADE level for certainty of evidence: ‘high’ indicates that we are very confident that the true effect lies close to that of the estimate of the effect; ‘moderate’ indicates that we are moderately confident in the effect estimate (the true effect is likely to be close to the estimate of the effect, but there is a possibility that it is substantially different); ‘low’ indicates that our confidence in the effect estimate is limited (the true effect may be substantially different from the estimate of the effect); and ‘very low’ indicates that we have very little confidence in the effect estimate (the true effect is likely to be substantially different from the estimate of effect)

Weight loss outcomes from published meta-analyses are presented in Figs. 2, 3 and ESM Table 11.

#### ***LCDs***

Ten meta-analyses reported on LCDs compared with higher-carbohydrate diets. Not all reported whether source RCTs were ad libitum or hypocaloric prescriptions, with results often pooled from both trial types. Definitions of LCDs varied, including <130 g/day, and <26% or <45% of energy intake from carbohydrate. Duration of interventions ranged from 8 weeks to 4 years.

Four high-quality meta-analyses [37–40] reported that LCDs and higher-carbohydrate diets were equally effective for weight loss, with mean difference ranging between <1 and <2.5 kg, at all durations. GRADE assessment ranged from low to high certainty of evidence. Just one meta-analysis reported greater weight loss with LCD, by 3.5 kg, using complete case data for pooled results [41]. The remaining critically low- to low-quality meta-analyses showed differences of <1 kg between the two diets [26, 27, 31, 33, 34].

Very low-carbohydrate diets (21–70 g of carbohydrate daily) showed no greater weight loss than higher-carbohydrate diets over durations of 3–36 months (weight mean difference [WMD] −0.7 kg; 95% CI −2.0, 0.7; *I*^2^ = 46%, *p* = 0.10) in a subgroup analysis [37]. A subgroup analysis of RCTs with low risk of bias reported no difference (WMD 0.9 kg; 95% CI -1.9, 3.6), while RCTs with high risk of bias showed greater weight loss for LCDs than higher-carbohydrate diets (WMD −1.8 kg; 95% CI −2.8, −0.7) [37].

#### ***High-protein diets***

All meta-analyses (*n* = 3) of high-protein diets were of critically low quality [26, 28, 30]. Critical domains unmet were presence of a review protocol and assessing risk of bias in synthesised findings (ESM Table 3). Only one provided a definition of ‘high protein’ (>20% of energy intake), reporting significantly greater weight loss (−1.2 kg; 95% CI −2.17, −0.24; *I*^2^ = 5%, *p* = 0.38) than with lower-protein diets (<20% of energy from protein) [28].

#### ***Mediterranean diets***

Two meta-analyses, of low and critically low quality, considered weight loss from Mediterranean diets [26, 32]. The control interventions combined no diet (usual care) and specified diets, including LFD and LCD. Pooled results indicated significantly greater weight loss with Mediterranean diets than in control groups, by 0.3 kg (low quality; [32]) to 1.8 kg (critically low quality; [26]), over durations of 4–24 weeks. A network meta-analysis also reported that Mediterranean diets were marginally more effective than LFDs for weight loss (−1.2 kg; 95% CI −1.99, −0.37; four RCTs, low quality; *p*-heterogeneity = 0.08; ESM Table 12) [25].

#### ***Formula meal replacement***

One high-quality meta-analysis [42] of nine RCTs including 931 participants reported that replacing one to three main meals daily (replacing 13–47% of total energy) produced significantly greater weight loss than low-energy diets over 12–52 weeks (−2.4 kg; 95% CI −3.3, −1.4; *I*^2^ = 84%, *p* < 0.001; GRADE moderate certainty of evidence).

#### ***VLEDs***

One high-quality meta-analysis [43] of two RCTs reported that VLEDs (1.7–2.1 MJ/day for 8–12 weeks) achieved greater weight loss at 3 months (−6.6 kg; 95% CI −9.5, −3.7; *I*^2^ = 58%, *p* = 0.12) and at 6 months (−5.7 kg; 95% CI −11.1, −0.4; *I*^2^ = 58%, *p* = 0.12), compared with an energy-restricted diet (4.2–6.3 MJ/day). These data were from participants who completed the trials.

#### ***High-monounsaturated-fatty-acid, vegetarian and low-glycaemic-index diets***

High-monounsaturated-fatty-acid (MUFA) [29] and vegetarian diets [35] showed greater weight losses, by −1.6 to −2 kg, than the control diets. Low-glycaemic-index diets [26, 36] were not associated with greater weight loss than control diets. Published meta-analyses of these diets were of low to critically low quality.

#### ***Intermittent fasting***

We did a post hoc analysis to evaluate all systematic reviews without meta-analyses (no pooled weight loss; *n* = 10) that were excluded from our main analysis (as intended protocol). Eight were systematic reviews whose source RCTs were already pooled in meta-analyses identified in this umbrella review. The remaining two systematic reviews compared altered eating patterns with conventional energy-restricted diets (ESM Table 13) [44, 45]. From these two reviews, three RCTs were identified: two for 5:2 diets reported no difference in weight loss (high-risk-of-bias RCTs) [46, 47], and one for time-restricted dieting reported 1.4 kg greater weight loss than conventional energy restriction (high-risk-of-bias RCT) [48].

#### ***Adherence***

Some meta-analyses offered assessed dietary adherence separately from weight change. Adherence assessed up to 1 year was poorer with very low-carbohydrate diets (<50 g of carbohydrate) than with LCDs (<130 g of carbohydrate) [34, 37, 40], possibly because most of the trials allowed increased carbohydrate intake for later weight loss maintenance. High adherence to VLEDs (up to 6 months), judged from rapid early weight loss and dietary assessment, led to better long-term results [43].

#### Effects of weight-loss diet intervention on HbA_1c_

Among published meta-analyses, HbA_1c_ reduction broadly followed weight loss, and differences between diet types assessed over 3–12 months were small. The published data do not permit an individual-level regression analysis to quantify weight loss-independent effects on HbA_1c_ (ESM Results, ESM Table 14).

### (2) Systematic review of intervention studies (either RCTs or NRSs) of diets for remission of type 2 diabetes

#### Identification of studies

From 373 records identified, we included 16 papers for data synthesis and quality assessment (ESM Fig. 2; excluded studies with reasons in ESM Table 5). These reported on 14 studies (six RCTs, eight NRSs), of seven diet types: total diet replacement (*n* = 4), formula meal replacement (*n* = 2), VLED (*n* = 2), very low-carbohydrate ketogenic diet (*n* = 1), Mediterranean diet (*n* = 2), LFD (*n* = 4) and the ADA diet (*n* = 1). Five studies compared diet interventions with usual care according to clinical guidelines, without providing foods or dietary products for participants [49–53]. Among these, three provided diabetes education or advice (Table 2) [49, 50, 52]. Included studies were conducted in Barbados, India, Italy, Qatar, South Africa, Spain, Thailand, the UK and the USA. Detailed characteristics and methodological quality are in the ESM Results, ESM Tables 15–17 and ESM Fig. 3.

**Table 2:** Type 2 diabetes remission (%) and mean weight loss (kg) from baseline according to different dietary regimens/patterns

Authors, yr (study)	Design	Diet INT	CON arm	Analysis and dropout during INT	T2D remission		Weight change (kg or %)		Risk of bias^a^	Funding
					INT	CON	INT	CON		
TDR										
Taheri et al. 2020 (DIADEM-I) [53]	RCT	TDR 3.3–3.4 MJ/d (800–820 kcal) for 12 wk, then food re-introduction over 12 wk (*n* = 70)	Usual care: no diet (*n* = 77)	ITT Dropout: INT = 15/70 (21%); CON = 10/77 (13%)	1 yr: 61% (43/70)	1 yr: 12% (9/77)	1 yr: −12.0	1 yr: −4.0	Low	Qatar National Research Fund
Lean et al. 2018 and 2019 (DiRECT) [51, 89]	RCT	TDR 3.5–3.6 MJ/d (825–853 kcal) for 12 wk, then food re-introduction over 2–8 wk. (*n* = 149)	Usual care: no diet (*n* = 149)	ITT Dropout 1 yr: INT = 32/149 (21%); CON = 0/149 Dropout 1–2 yr: INT = 16; CON = 0	1 yr: 46% (68/149) 2 yr: 36% (53/149)	1 yr: 4% (6/149) 2 yr: 3% (5/149)	1 yr: −10.0 2 yr: −7.6	1 yr: −1.0 2 yr: −2.3	Low	Diabetes UK
Bynoe et al. 2020 [102]	Single arm	TDR 3.2 MJ/d (760 kcal) for 8 wk, then food re-introduction over 4 wk (*n* = 25)	N/A	ITT Dropout:1/25 at 8 mo	8 wk: 60% (15/25) 8 mo: 36%^b^ (9/25)	N/A	8 wk: −10.1 8 mo: −8.2	N/A	Critical	A grant from Virgin Unite
Steven et al. 2016 [103]	Single arm	TDR 2.6–2.9 MJ/d (624–700 kcal) for 8 wk, then food re-introduction over 2 wk. (*n* = 30)	N/A	ITT Dropout: 1 at 1 wk due to not meeting weight loss target	8–10 wk: 40% (12/30) 8 mo: 43% (13/30)	N/A	8–10 wk: −14.2 6 mo: −13.3	N/A	Critical	NIHR Newcastle
Formula meal replacement										
Gregg et al. 2012 (Look AHEAD) [49]	RCT	Liquid meal replacement to achieve goal of 5.0–7.5 MJ/d (1200–1800 kcal) with two meal replacements during 0–20 wk and then one meal replacement thereafter (*n* = 2262)	Usual care: diabetes support and education; no diet (*n* = 2241)	ITT: ancillary analysis Dropout 1 yr: INT = 74/2570 (3%); CON = 112/2575 (4%)	1 yr: 11.5% (247/2157) 2 yr: 10.4% (218/2090) 3 yr: 8.7% (181/2083) 4 yr: 7.3% (150/2056)	1 yr: 2.0% (43/2170) 2 yr: 2.3% (48/2101) 3 yr: 2.2% (46/2085) 4 yr: 2.0% (41/2042)	1 yr: −8.6%	1 yr: −0.7%	Some concerns	US Department of Health and Human Services and NIH
Mottalib et al. 2015 (Why WAIT) [57]	Single arm	Liquid meal replacement for breakfast and lunch to achieve goal of 5.0–7.5 MJ/d (1200–1800 kcal), 40% CHO, 30% fat, 30% protein (*n* = 126)	N/A	ITT: ancillary analysis Dropout: 38/126 (30%) at 1 yr	1 yr: 3.2%^c^ (4/126)	N/A	1 yr: −7.2 in those achieving remission	N/A	Critical	See footnote^d^
Mediterranean diets and LFDs										
Gutierrez-Mariscal et al. 2021 [54]	RCT	Mediterranean diet No E restriction (*n* = 80)	LFD No E restriction (*n* = 103)	Complete case analysis in subset of people with CHD with T2D in original trial. Ancillary analysis	5 yr: 41.3% (33/80)	5 yr: 38.8% (40/103)	5 yr: −1.16	5 yr: −1.4	Some concerns	See footnote^e^
Esposito et al. 2014 [59]	RCT	Mediterranean diet E restriction Women: 6.3 MJ/d (1500 kcal) Men: 7.5 MJ/d (1800 kcal) (*n* = 108)	LFD E restriction Women: 6.3 MJ/d (1500 kcal) Men: 7.5 MJ/d (1800 kcal) (*n* = 107)	ITT: ancillary analysis Dropout 1 yr: INT = 10/108 (9%); CON = 10/107 (9%)	1 yr: 14.7% (15/102) 2 yr: 10.6% (9/85) 3 yr: 9.7% (7/72) 4 yr: 7.7% (4/52) 5 yr: 5.9% (2/34) 6 yr: 5.0% (1/20)	1 yr: 4.1% (4/97) 2 yr: 4.7% (3/64) 3 yr: 4.0% (2/50) 4 yr: 2.9% (1/35) 5 yr: 0 6 yr: 0	1 yr: −6.2	1 yr: −4.2	Some concerns	Second University of Naples
Mollentze et al. 2019 [52]	Pilot RCT	LFD^f^ E restriction, mainly vegetables and soups (*n* = 9)	Usual care: diet advice (*n* = 9)	ITT No dropout	3 mo: NR 6 mo: 22.2% (2/9)	3 mo: NR 6 mo: 0%	3 mo: −9.0% 6 mo: −9.6%	3 mo: −1.9% 6 mo: −1.5%	High	Mr Christo Strydom, South Africa
Sarathi et al. 2017 [104]	Single arm	LFD 6.3 MJ/d (1500 kcal) (*n* = 32)	N/A	ITT No dropout	1 yr: 75.0% (24/32) 2 yr: 68.8% (22/32)	N/A	NR	N/A	Critical	No funding
Dave et al. 2019 [105]	Single arm	LFD (ADA diet^g^) (*n* = 45)	N/A	ITT Dropout: 4 at 5y	1 yr: 71.1% (32/45) 5 yr: 42.2%^h^ (19/45)	N/A	1 yr: −7.6 5 yr: −6.4	N/A	Critical	No funding
Ketogenic diet										
Hallberg et al. 2018 and Athinarayanan et al. 2019 (VIRTA) [50, 55]	Non-RCT	VLCKD CHO <30 g/d to achieve ketosis, 1.5 g/kg protein per d, 3–5 servings of non-starchy vegetables, multivitamin, vitamin D_3_ and *n*-3 fatty acids supplements No E restriction advised (*n* = 262)	Usual care: local medical provider and education (*n* = 87)	ITT: ancillary analysis Dropout 1 yr: INT = 44/262 (17%); CON = 9/87 (10%) Dropout 1–2 yr: INT = 24; CON = 10	1 yr: 19.8%^i^ (52/262) 2 yr: 17.6% (46/262)	1 yr: NR 2 yr: 2.3% (2/87)	1 yr: −13.8 2 yr: −11.9	1 yr: +0.6 2 yr: +1.3	Serious	Virta Health
VLED										
Umphonsathien et al. 2019 [56]	Single arm	VLED 8 wk 2.5 MJ/d (600 kcal) food-based diet, then food re-introduction over 4 wk (*n* = 20)	N/A	ITT Dropout: 1 during run-in	8 wk: 75% (15/20) 12 wk: 75% (15/20)	N/A	8 wk: NR 12 wk: −9.5	N/A	Critical	Prasert Prasarttong-Osoth Research Fund
Thomas and Shamanna, 2018 [60]	Single arm	VLED 1 wk 2.9 MJ/d (700 kcal) food-based on diet, then advice diet for ideal body weight (*n* = 9)	N/A	ITT Dropout: 1 after completing E restriction phase	1 yr: 22.2%^j^ (2/9)	N/A	1 yr: −4.2	N/A	Critical	NR

Remissions in Gregg et al. 2012 [49 and Esposito et al. 2014 [59] are prevalence estimates with raw cases/denominators.

^a^Cochrane Risk of Bias tool version 2 for RCT, and Risk Of Bias In Non-randomised Studies – of Interventions for non-RCT and single-arm intervention

^b^ITT analysis was calculated from nine participants, who had fasting plasma glucose <7 mmol/l and no medication, in a total of 25 participants. For completer analysis, remission rate was 37.5% calculated from nine out of 24 completers at 8 months

^c^ITT analysis calculated from four out of 126 participants who had HbA_1c_< 48 mmol/mol (<6.5%) and no medication at 1 year. For completer analysis, remission rate was 4.6% calculated from 52 out of 88 completers

^d^Why WAIT programme received contributions from Novartis Medical Nutrition (currently Nestlé HealthCare Nutrition) and LifeScan.

^e^Ministerio de Economia y Competitividad & the Instituto de Salud Carlos III of Spain, the Directorate General for Assessment and Promotion of Research and the European Union's (EU's) European Regional Development Fund

^f^See ESM Table 15 for details

^g^Diet according to the recommendation of the ADA [66]

^h^ITT analysis was calculated from 19 participants who achieved remission in a total of 45 participants. For completer analysis, remission rate was 46.3% calculated from available data at 12 months (19 out of 41 completers)

^i^ITT analysis calculated from 52 out of 262 participants in the intervention group who had HbA_1c_< 48 mmol/mol (<6.5%) and no medication at 1 year. For completer analysis, remission rate was 26% calculated from available data at 12 months (52 out of 204 completers)

^j^ITT analysis calculated from two participants who had HbA_1c_< 48 mmol/mol (<6.5%) and no medication at 1 year, in a total of nine participants. For completer analysis, remission rate was 25% calculated from available data at 12 months (two out of eight completers)

CON, control; d, day; DIADEM-I, Diabetes Intervention Accentuating Diet and Enhancing Metabolism-I; E, energy; INT, intervention; Look AHEAD, Action for Health in Diabetes; mo, month; N/A, not applicable; NIH, National Institutes of Health; NIHR, National Institute for Health Research; NR, not reported; T2D, type 2 diabetes; TDR, total diet replacement; VIRTA, Virta Health Corp; VLCKD, very low-carbohydrate ketogenic diet; Why WAIT, Weight Achievement and Intensive Treatment; wk, week; yr, year

#### Definition of type 2 diabetes remission

All included studies defined remission as a diagnostic test result, without glucose-lowering medication, below the WHO threshold for diagnosis of type 2 diabetes (HbA_1c_ < 48 mmol/mol [6.5%], or fasting plasma glucose <7 mmol/l), but they differed in the duration prior to assessment of remission (ESM Tables 15–16). Some studies [54–57] subdivided results as previously proposed by Buse et al. [58]. Glucose-lowering medications were not routinely withdrawn at the beginning of diets in some of the studies, so only minimum remissions can be reported.

#### Effects of diets on type 2 diabetes remission and weight at 1 year

**Fig. 4:**
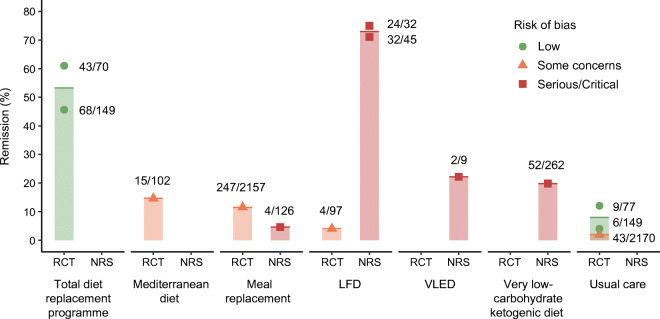
Percentage of remissions of type 2 diabetes at 12 months after intervention with different diet types, stratified by study design and risk of bias. Each dot, with varying shapes to reflect risk of bias, indicates the data point for each of the studies mentioned in the main text which provided data in this form at 12 months. The column represents the mean for the diet type. Remission was defined as either HbA_1c_ < 48 mmol/mol (<6.5%) or fasting plasma glucose <7 mmol/l, with no glucose-lowering medication. Total diet replacement programmes included an initial low-energy formula diet, prescribed for an 8–12 week induction phase, followed by stepped food re-introduction aimed to achieve energy balance for weight loss maintenance. VLED advised a 2.9 MJ (700 kcal) food-based diet for 1 week, then dietary advice for energy intake that matched for ideal body weight. Very low-carbohydrate ketogenic diet was ad libitum intake, carbohydrate <30 g/day to achieve ketosis and 3–5 servings of non-starchy vegetables. Usual diet or standard diet interventions included diabetes education support, but no new diet intervention

Remission rates and weight changes at 1 year are summarised in Fig. 4 and Table 2, with GRADE certainty of evidence in Table 3.

**Table 3:** Summary of findings of type 2 diabetes remission at 1 year after diet intervention compared with baseline with GRADE certainty of a body of evidence

Diet	Conclusion statement	No. of participants (no. of studies)	Certainty in the evidence^a^	Comments
TDR	TDR leads to a large increase in T2D remission by a median of 54% from baseline (range 46–61%), when compared with standard care (4–12%).	445 (2 RCTs)	⊕⊕⊕⊕ HIGH	Low-risk-of-bias RCTs, pre-specified outcomes with power calculation
Meal replacement	Meal replacement likely leads to T2D remission by 11% from baseline, when compared with standard care plus diabetes education (2%).	4503 (1 RCT)	⊕⊕◯ MODERATE Due to possible publication bias	Ancillary observational analysis of RCT
Mediterranean diet	Mediterranean diet may lead to T2D remission by 15% from baseline, when compared with LFD (4%).	215 (1 RCT)	⊕⊕◯◯ LOW Due to imprecision^b^ and possible publication bias	Small sample size, and ancillary observational analysis of RCT
Very low carbohydrate ketogenic diet	The evidence is very uncertain about the effect of ketogenic diet on T2D remission due to serious risk of bias of the study methods and imprecision, although one non-RCT reported a remission rate of 20%, compared with no remission in usual care with diabetes education.	349 (1 non-RCT)	⊕◯◯◯ VERY LOW Due to serious risk of bias (rated down 2 levels) and imprecision^b^	Lack of randomisation, uncontrolled confounding, selection bias, incomplete outcome data, possible selective reporting, imprecision and imbalance between groups
VLED (food based)	The evidence is very uncertain about the effect of food-based VLED on T2D remission, although one small uncontrolled intervention study reported a remission rate of 22%.	9 (1 single group uncontrolled intervention)	⊕◯◯◯ VERY LOW Due to critical risk of bias (rated down 3 levels), imprecision and potential publication bias	Lack of randomisation, uncontrolled confounding, selection bias and selective reporting of result. Only one positive, small study

Remission is defined as either HbA_1c_ < 48 mmol/mol (<6.5%) or fasting plasma glucose <7 mmol/l and no glucose-lowering medication

^a^GRADE level for certainty of evidence: ‘high’ indicates that we are very confident that the true effect lies close to that of the estimate of the effect; ‘moderate’ indicates that we are moderately confident in the effect estimate (the true effect is likely to be close to the estimate of the effect, but there is a possibility that it is substantially different); ‘low’ indicates that our confidence in the effect estimate is limited (the true effect may be substantially different from the estimate of the effect); and ‘very low’ indicates that we have very little confidence in the effect estimate (the true effect is likely to be substantially different from the estimate of effect)

^b^Rated down one level due to imprecision, as the sample size is less than an optimal information size of 400

T2D, type 2 diabetes; TDR, total diet replacement

Programmes that included an induction phase of formula ‘total diet replacement’ were studied in two RCTs with low risks of bias. Compared with remissions of 4–12% in well-matched usual care control arms, the interventions generated median 54% remission at 12 months from baseline (*N* = 445, two RCTs; GRADE high certainty of evidence), with diabetes durations <6 or <2 years, and mean weight loss of 10 and 12 kg. These two RCTs were designed with remission as the primary outcome [51, 53].

Among trials reporting post hoc analyses for remission, one using two meal replacements/day during 0–20 weeks and one per day thereafter reported 11% (247/2157) remission at 1 year (prevalence estimates), with mean weight loss 8.6 kg, compared with 2% (43/2170) in standard care (*N* = 4503; GRADE low certainty of evidence), with some concern over risk of bias [49].

A single RCT of Mediterranean diet over 12 months reported a remission prevalence of 15% (15/102), with mean weight loss 6.2 kg, compared with 4% (4/97) with weight loss 4.2 kg in the control LFD arm (*N* = 215; GRADE low certainty of evidence; some concern over risk of bias) [59].

No RCT has evaluated LCDs/ketogenic diets for type 2 diabetes remission. A non-randomised, controlled study of a very low-carbohydrate (ketogenic) diet reported 20% remission (52 out of 262 who started treatment [ITT] who had HbA_1c_ < 48 mmol/mol [6.5%] without diabetes medication), with mean weight loss 13.8 kg, at 1 year, compared with no remission in a control arm (*N* = 349; GRADE very low certainty of evidence; serious risk of bias) [50]. The dropout rate was 17% (44/262) and 22% had incomplete outcome data. This study primarily focused on HbA_1c_ lowering, not remission, so glucose-lowering medications were not routinely withdrawn.

Another very small uncontrolled study evaluated a 1 week 2.9 MJ/day (700 kcal) food-based diet, finding 22% (2/9) remission at 1 year, with mean weight loss 4.2 kg (*N* = 9, one single-arm intervention; GRADE very low certainty of evidence; critical risk of bias) [60].

#### Sources of heterogeneity

Single-arm intervention studies reported higher remission than RCTs. Participants with shorter type 2 diabetes duration, and Asian ethnicity, were more likely to achieve remission than those with longer type 2 diabetes duration or another ethnicity (Table 2, ESM Tables 15,16).

## Discussion

### Dietary weight reduction for people with type 2 diabetes

This study was conducted to inform practice and policy over dietary advice for weight management of people with type 2 diabetes. It has therefore focused on interventions in free-living individuals, with a view to long-term management. Based on methodological quality and certainty of the evidence, our umbrella review of meta-analyses found that VLEDs and formula meal replacements produce greater weight losses than conventional low-energy diets. The evidence does not favour LCDs above higher-carbohydrate diets, nor other dietary approaches, i.e., high-protein, Mediterranean, high-MUFA, vegetarian and low-glycaemic-index diets, above control diets. Currently popular intermittent fasting was only captured in systematic reviews without meta-analysis (high-risk-of-bias RCTs) [44, 45]. The evidence, albeit of variable ‘quality’, is rather consistent such that no one diet type is superior over others for weight management in type 2 diabetes.

While the evidence does not suggest important differences between macronutrient compositions in effectiveness, there may be differences in cost-effectiveness. The evidence on relative cost-effectiveness of weight-loss diet programmes is limited from head-to-head diet comparison trials, but one RCT showed that LCD was not more cost-effective than the standard weight-loss diet [61]. In the Doctor Referral of Overweight People to Low Energy total diet replacement Treatment (DROPLET) trial among people without diabetes, in routine practice, a total diet replacement programme (formula diets) with behavioural support proved more cost-effective than nurse-led dietary advice for long-term prevention of obesity-related diseases [62]. For diabetes remission, a total diet replacement programme was estimated to be both more cost-effective and cost-saving than standard care in the DiRECT trial, reflecting reduced need for medications and fewer diabetes complications [63].

Health benefits from weight management depend largely on long-term control of body weight. Most of the evidence cited relates to short-term outcomes, relevant to the initial weight loss induction phase of weight management. Few trials have reported data beyond 12 months, to reflect weight loss maintenance, which may demand different behavioural strategies. One large RCT of high-protein diet suggested benefit for weight loss maintenance, increasing satiety and energy expenditure, albeit for a maintenance phase of only 6 months after completing weight loss [64]. Nutrient-specific effects have been postulated, but are likely to be overwhelmed by variable behavioural responses to dietary advice [65]. Behavioural programmes help to sustain new behaviours, relationships with foods and adherence to dietary advice [66–68]. Consistent evidence is also accruing that long-term weight loss maintenance is better after more rapid early weight loss [69]. Thus, treatments effective for weight loss only in the short term may have long-term value if complemented with a good weight loss maintenance strategy. Practitioners can therefore be confident that a variety of diet types can all achieve the intended weight losses, and potentially remissions of type 2 diabetes, if their patients are able to adhere to the programme sufficiently.

The analyses contradict some popular claims about specific diets: in particular, ‘low-carb’ diets hold no overall advantage for weight loss when compared with higher-carbohydrate diets. However, we cannot conclude that any individual with type 2 diabetes, in any context, will do equally well with any diet advice, or that a skilled practitioner may not have greater success advising one diet type. The skills and empathy of practitioners may overcome any diet-specific effects on weight loss by providing consistent evidence-based support [70]. Realistic trials are required, in which individuals are offered choices, perhaps using *n* = 1 randomised trial designs.

### Weighing benefits against risks

Food is fundamental for personal and social wellbeing, and diets can be psychologically testing. Patient preferences, culture, context and lifestyle demand open conversation and shared decision making between practitioners and patients. For either medication or diet, weighing benefits against risks is vital: treatment benefits are often overestimated but harms underestimated [71]. Although all diet types are similarly effective for weight control, health risks were not systematically reported across the studies, and could differ [72]. More rapid early weight loss with more intensive programmes is associated with better longer-term weight outcomes [69], but severe caloric restriction without attention to nutrient content can have unwanted effects. Blood pressure falls with weight loss, and postural hypotension, common in older people and those with diabetes, is aggravated during rapid weight loss if diuretic or antihypertensive drugs are taken concurrently [73]. Hypoglycaemia is possible if hypoglycaemic drugs are also taken [74]. Diets other than nutritionally complete formula diets could incur vitamin and mineral deficiencies [75]. With ketogenic diets, heart failure and neurological problems from thiamine deficiency have been reported [76, 77], as well as reduced intakes of folate, iron and magnesium [12]. Replacing high-carbohydrate foods with red or processed meat (high animal protein and fat) increases sodium and long-chain saturated fat intakes, elevating LDL-cholesterol [15, 16] and potentially increasing cardiovascular disease risk [78–80]. High protein intake has been associated with kidney diseases in several observational studies [81]. Metabolic ketoacidosis with ketogenic diets is a hazard, particularly with sodium−glucose cotransporter 2 (SGLT2) inhibitors [82–87]. Meanwhile, extreme fat avoidance provokes cholelithiasis [88].

### Remission of type 2 diabetes

Current evidence on diets for type 2 diabetes remission is more limited. Only two RCTs had remission as the pre-specified outcome, both relatively large and using almost identical designs and diets, with very similar results, but in very different populations, notably with different durations of diabetes [51, 53, 89]. A large majority can achieve remission if they maintain sufficient weight loss.

NRSs (non-RCT, single-arm intervention) reported remission rates ranging from 3% to 75% by ITT, over various follow-up durations. The highest remission rates, up to 75%, were in people with newly diagnosed diabetes or with <2 years of type 2 diabetes duration. Much lower 20–22% remissions were reported with longer type 2 diabetes duration (8 years) or a very brief diet period (1 week). However, these studies did not all fully ascertain remission status, and they had critical risks of bias due to lack of comparator groups and/or randomisation. NRSs reflect performance among those who select and can adhere to a particular diet, and so usually reported better results than those featuring random assignment. In some cases, remission rates were reported for completers only, rather than using the ITT population to properly guide healthcare practice and policy. Despite extracting baseline data for ITT analysis, residual bias/confounding may remain with these study designs.

The main contributor to HbA_1c_ reduction and remission appears to be weight loss, irrespective of diet type. From the high-quality studies with high GRADE certainty, structured programmes with an intensive induction phase with total diet replacement were effective. Remission of diabetes occurs when a patient no longer satisfies the diagnostic criteria, without receiving glucose-lowering medication. To ascertain remission for those already prescribed glucose-lowering drugs, a therapeutic trial of withdrawing medication is necessary, with an appropriate protocol for re-introduction if necessary. Confirmation over a defined duration (e.g., 6 or 12 months) will be required for re-classifying individuals, and for legal or insurance purposes. The diagnostic HbA_1c_ cut-off for diabetes of 48 mmol/mol (6.5%) was defined by WHO as broadly the level where diabetes-specific microvascular complications start to emerge [90, 91]. However, many people in remission from type 2 diabetes remain in the pre-diabetes range of HbA_1c_, where cardiovascular disease risk begins to rise [92, 93]. Lowering HbA_1c_ to very low levels with multiple medications among people with longstanding disease is associated with increased mortality rate, possibly by relative hypoglycaemia provoking arrythmias [94]. No such concerns have been reported in the small numbers who achieved and sustained HbA_1c_ < 42 mmol/mol (<6.0%) from diet restriction [89].

Most type 2 diabetes is treated in primary care, the setting for both published remission trials using an intensive ‘total diet replacement’ induction phase with formula diets [51]. Simpler food-based programmes may be effective. A service evaluation from one UK general practice reported weight loss and remission in 59 out of 128 patients who opted for, and persisted with, LCD advice for a mean 23 months [95]. This completers’ analysis omits information about numbers who declined the diet, who started but failed to persist and who did not provide outcome data at designated times. The LCD was routinely offered since 2013, and the total number of patients with type 2 diabetes was 473 at the time of evaluation, so these data imply that 12.5% of the practice achieved remission [95]. A population-based cohort study from 49 general practices in the UK (the Anglo–Danish–Dutch Study of Intensive Treatment in People with Screen-Detected Diabetes in Primary Care [ADDITION-Cambridge]) included 867 participants with newly diagnosed type 2 diabetes; after being followed up for 5 years, there was an overall 30% remission (*n* = 257/867; ITT analysis). Loss of >10% of baseline body weight in the first year after diagnosis was associated with 70% higher chance of remission at 5 years [96]. Every 1 kg of weight loss was associated with 7% higher chance of remission at 5 years, regardless of specific diet regimens or lifestyle interventions [96]. There is therefore consistent evidence that remission should be attempted as early as possible from diabetes diagnosis [70, 96].

### Limitations

AMSTAR 2 assesses the quality of meta-analyses, prioritising critical domains, where errors and bias can impact pooled findings (ESM Table 2). Only one or two flaws can label a meta-analysis ‘low’ or ‘critically low’, with some criteria potentially subjective (e.g., adequacy of the literature search; ESM Table 2). In the umbrella review, many meta-analyses were of ‘low’ and ‘critically low’ AMSTAR 2 quality, predominantly through ‘no protocol reported’ (despite clear and sound methods) and no assessment of publication bias. Many meta-analyses had fewer than ten RCTs to permit assessment of publication bias by funnel plot [97]. If AMSTAR 2 criteria are relaxed for protocol reporting and publication bias, the meta-analyses allow some confidence in the consistent findings of little/no difference in weight loss between any diets.

Although the search strategy was wide and not language-restricted, most studies included European participants; results may not be equally applicable to other ethnic and/or deprived communities. South Asians develop type 2 diabetes at younger ages, more rapidly and with lower BMI, so may be more sensitive to weight loss, with physiological differences in insulin resistance, body composition and fat oxidation [98, 99].

The criteria used in the reported meta-analyses and studies focused on specific diet types. However, not all reported sufficient detail about macronutrient or micronutrient contents, or prescribed and reported energy intakes, including energy intake of nutrient-restricted ad libitum diets, which limits interpretation and transferability of results. Control diets used in the meta-analyses and source RCTs also varied, including ‘usual’ diets in different countries, as well as specified dietary regimens (Table 1). Despite this, differences in weight loss between intervention and control diets, commonly 0–2 kg, are of little clinical significance. Durations of interventions varied: as weight regain is frequent over a longer period, heterogeneity might be expected. However, duration did not introduce heterogeneity, probably because trials with longer follow-up tended to be evaluating more intensive interventions with greater initial weight loss, such that the net weight changes at endpoint are similar to short-term trials.

Given the extent of literature concluding that differences in weight control or HbA_1c_ from different diet compositions are not clinically significant, future trials of similar diet comparisons are unlikely to add useful information. Instead, evidence from clinical practice is needed to identify safe and effective approaches to achieve and maintain weight loss with available skills and training, to assess long-term outcomes from high-quality trials and prospective audits of practice with different diets. Interpretating the existing data might be enhanced through individual patient data meta-analysis. Alternatively, the very large amount of work entailed in conducting repeated meta-analyses, and the limitations of different inclusion criteria and detailed methods, support a prospective meta-analysis approach [100, 101]. All primary studies for inclusion should use an RCT design, with data analyses conducted ‘blind’. They should define the intervention clearly (e.g., diets, physical activity, and behavioural and psychological support), and address separately the induction (usually 3–6 months) and maintenance (≥12 months) phases of weight management, potentially employing different methods within a treatment programme.

## Supplementary Information

ESM 1 (PDF 815 kb)

## Data Availability

All data analysed during this work are included in this published article and its ESM file, and in the relevant references.

## Abbreviations

AMSTAR: A Measurement Tool to Assess Systematic Reviews
DiRECT: Diabetes Remission Clinical Trial
GRADE: Grading of Recommendations, Assessment, Development and Evaluations
ITT: Intention to treat
LCD: Low-carbohydrate diet
LFD: Low-fat diet
MUFA: Monounsaturated fatty acid
NRS: Non-randomised study
VLED: Very low energy diet
WMD: Weight mean difference
