# Supplementary material for: Diets for weight management in adults with type 2 diabetes: an umbrella review of published meta-analyses and systematic review of trials of diets for diabetes remission
Source: Diabetologia. 2021 Nov 17;65(1):14–36. doi: 10.1007/s00125-021-05577-2 (PMC8660762; doi:10.1007/s00125-021-05577-2)
Supplement: Supplementary file 1 — (PDF 815 kb) [file 125_2021_5577_MOESM1_ESM.pdf]

Electronic Supplementary Material

Diets for weight management in adults with type 2 diabetes: an umbrella review of published meta-analyses and a systematic review of trials of diets for diabetes remission

Dr Chaitong Churuangsuk, MD, PhD [1, 2] - https://orcid.org/0000-0003-4373-6395

Mr Julien Hall, MSc [1] - https://orcid.org/0000-0003-0597-9513

Dr Andrew Reynolds, PhD [3,4] - https://orcid.org/0000-0001-8952-3892

Professor Simon J. Griffin, DM [5,6] https://orcid.org/0000-0002-2157-4797

Dr Emilie Combet, PhD [1] - https://orcid.org/0000-0002-9302-8971

Professor Michael E.J. Lean, MA, MB, BChir, MD, FRCP, FRSE [1]* - https://orcid.org/0000-0003-2216-0083

[1] Human Nutrition, School of Medicine, Dentistry and Nursing. College of Medical, Veterinary and Life Sciences, University of Glasgow, Glasgow, UK.

[2] Division of Internal Medicine, Faculty of Medicine, Prince of Songkla University, Hat Yai, Thailand.

[3] Department of Medicine, University of Otago, Dunedin, Otago, New Zealand.

[4] Edgar National Centre for Diabetes and Obesity Research, University of Otago, Dunedin, Otago, New Zealand.

[5] MRC Epidemiology Unit, Institute of Metabolic Science, School of Clinical Medicine, University of Cambridge, Cambridge, UK.

[6] Primary Care Unit, Department of Public Health and Primary Care, School of Clinical Medicine, University of Cambridge, Cambridge, UK.

***Corresponding author:**

Prof Michael E. J. Lean

Room 2.19, Level 2, New Lister Building, 10-16, Alexandra Parade, Glasgow Royal Infirmary, Glasgow, G31 2ER.

E-mail: mike.lean@glasgow.ac.uk

ESM Methods

1) Additional details on methods of an umbrella review of published meta-analyses of diets for weight loss in type 2 diabetes

Eligibility criteria

Papers were eligible if they met the following criteria:

a) Systematic reviews with meta-analyses of randomised controlled trials (RCT)
b) compared any type of diets with any control diets or usual/routine care.
c) adult participants, either sex, with type 2 diabetes
d) provided pooled results on a weight loss outcome (primary outcome) and/or changes in HbA_{1c} (secondary) as mean difference between the two diet interventions, or mean difference from baseline, at any length of follow-up
e) Papers were excluded if the diet intervention (or comparators) included additional components (e.g., drugs, bariatric surgery, exercise, or education).

Information source

We conducted a systematic literature search in electronic databases including Medline (OvidSP), PubMed, Web of Science Core Collection, Cochrane Database of Systematic Reviews from their inception to 4 February 2020, with an update on 07 May 2021.

We used free text and Mesh terms as follow: *diet, weight loss or weight reduction, type 2 diabetes, and meta-analysis*. Reference lists of included reviews were also searched. Neither search restrictions nor limits were applied. A full search strategy for Medline is available in ESM Table 1.

Selection process and data collection process

Two authors (CC and EC) independently screened titles and abstracts for eligible meta-analyses against the eligibility criteria. A consensus was reached out when there was disagreement between the two authors. Data extraction was performed by two authors independently (CC and JH).

Data items

The following data were extracted. Outcome variables: definitions of diet interventions and controls, pooled results on amount of weight loss and HbA_{1c} and 95% confidence interval, I-square heterogeneity statistic and its p-value, and duration of study. Other variables: authors, year, title, population characteristics, numbers of included trials and numbers of total participants from each meta-analysis, publication bias, and GRADE certainty of evidence (if GRADE was already applied by the meta-analysis authors).

Risk of bias (methodological quality) assessment

We used A Measurement Tool to Assess Systematic Reviews 2 (AMSTAR-2) to assess methodological quality (internal validity) of included meta-analysis.[1] Two authors (CC and JH) independently performed quality assessment. The tool is not intended to generate an overall score,[1] although several review authors had calculated the overall score to indicate methodological quality of the meta-analyses.[2] As suggested by the tool developers, critical domains, for which biases could seriously affect the validity of the pooled results, were identified for evaluating and classifying the quality of included meta-analyses (ESM Table 2-3). Overall methodological quality comprises four level (i.e., high, moderate, low, and critically low) based on the criteria specified in ESM Table 2, and interpreted as below:

- High quality - the meta-analysis provides an accurate and comprehensive summary of the results of the available studies that address the question of interest.
- Moderate - the meta-analysis has more than one weakness, but no critical flaws. It may provide an accurate summary of the results of the available studies.
- Low - the meta-analysis has a critical flaw and may not provide an accurate and comprehensive summary of the available studies that address the question of interest.
- Critically low - the meta-analysis has more than one critical flaw, and should not be relied on to provide an accurate and comprehensive summary of the available studies.

Reporting bias assessment

The publication bias of each meta-analysis was assessed by its authors, unless fewer than 10 RCTs were included, which makes publication bias assessment by funnel plot unreliable.

Certainty of evidence assessment

We used the Grading of Recommendations, Assessment, Development and Evaluations (GRADE) to rate the certainty of evidence from the included meta-analyses,[3] which indicates the confidence in a pooled result, in this case, weight loss by each diet. GRADE specifies four levels of certainty of evidence (i.e., high, moderate, low, and very low), derived from five domains: risk of bias of RCTs, consistency of outcomes, relevance to a research question, precision of effect size, and publication bias. If it had not been graded already by their authors, GRADE was applied by the umbrella review authors to the pooled result of those meta-analyses. For GRADE's imprecision domain, the umbrella review authors rated down for imprecision if the total sample size is less than 400 participants (an optimal information size, suggested by Guyatt *et al* [4]). If a process of conducting meta-analysis is valid, their pooled result is also valid and reliable. Full GRADE assessment is presented in ESM Table 4.

Among the meta-analyses that were graded by their authors, there is inconsistency for the imprecision domain for 2 meta-analyses: 1) van Zuuren et al 2018, 8-16 weeks result; and 2) Pfeiffer et al 2020 (ESM Table 4). For van Zuuren et al 2018, the authors gave the following reason: "We did not downgrade for imprecision. Although the minimal important difference is not established, we considered a reduction of <5% to be not

important. Therefore, the effect estimate is rather precise". Pfeiffer et al 2020 did not provide reason for not rating down for imprecision despite the total sample size was less than 400."

2) Additional details on methods of a systematic review of diets for type 2 diabetes remission

Eligibility criteria

We planned to include RCTs reporting remission of type 2 diabetes as the primary outcome, as this design provides the most trustworthy evidence to evaluate the effect of interventions. However, we anticipate that there will be too few RCTs of diets conducted primarily for type 2 diabetes remission to draw a definitive conclusion. Given the recent recognition that reversal of type 2 diabetes into a remission state is possible without bariatric surgery, to best inform clinical and policy decisions, we also included evaluations of non-randomised intervention studies (NRS) of diets on type 2 diabetes remission, which if well conducted might contribute to 'best available' advice from the totality of the evidence.[5,6]

According to the Cochrane handbook, NRS refers to several study designs, for example, controlled pre-post study, cohort study, case-control study, and so on, this would result in serious heterogeneity if all NRS designs are included for synthesis. In this systematic review, the criteria for inclusion of NRS are set as following:

- Non-randomised controlled trials
- Single arm interventions without a control group

In these types of NRS an intervention was provided to participants and outcomes were assessed at designated specific time points (baseline and at the end of intervention), however, due to the non-randomised design, they could exhibit selection bias and confounding bias due to the non-randomised design. We excluded observational studies of self-reported dieters.

All potential studies must report proportion/percentage/rate of type 2 diabetes remission, after dietary intervention. Studies were excluded if the population was not adults with type 2 diabetes, and/or no type 2 diabetes remission outcome was reported. Studies of diet plus other measures (i.e., drugs, and bariatric surgery) as a co-intervention were also excluded. Thus, papers were eligible if they met the following criteria:

a) RCTs comparing any type of diets with any control diets or usual/routine care, using either food-based or formula diets
b) NRS a as following: 1) non-randomised controlled trials, and 2) single arm intervention without control group of any type of diets.
c) reported proportion/percentage/rate of type 2 diabetes remission after dietary intervention
d) Studies were excluded if:
    a. diet intervention (or comparators) included additional components (e.g., drugs, bariatric surgery, exercise, or education)
    b. observational studies of self-reported dieters, without intervention provided

Information source

We conducted a systematic literature search in electronic databases including Medline (PubMed), Embase (OvidSP), and Cochrane Trial Registry (CENTRAL) from their inception to 4 August 2020, with an update on 10 May 2021.

We used free texts and MeSH terms as follow: *type 2 diabetes, remission, diet, and intensive lifestyle intervention*, with Boolean NOT for *surgery or bypass* in titles and abstracts. Reference lists of the meta-analyses from the umbrella reviews, and the studies included in this systematic review were also searched. Neither search restrictions nor limits were applied. A full search strategy for Embase is available in ESM Table 1.

Selection process and data collection process

Two authors (CC and EC) independently screened titles and abstracts for eligible studies against the eligibility criteria. A consensus was reached out when there was disagreement between the two authors. Data extraction was performed by two authors independently (CC and JH). EC was consulted where there was disagreement.

Data items

The following data were extracted. Outcome variables: diet interventions, duration of diets, definition of type 2 diabetes remission, percentage of remission, amount of weight loss, and methods of analysis (whether intention to treat or completer analysis). Other variables: authors, year, title, population characteristics including type 2 diabetes duration, dropout rate, and funding agency.

Risk of bias (methodological quality) assessment

To assess internal validity of study methodology, the Cochrane Risk of Bias tool 2.0 was used for RCT [7], and the Risk Of Bias In Non-randomised Studies - of Interventions (ROBIN-I) for NRS [8]. Risk of bias assessment was performed by CC and JH. EC was consulted where there was disagreement.

Reporting bias assessment

Given that this systematic review has been conducted without meta-analysis, the publication bias or reporting bias was assessed using the domain ‘Selection of the reported result’ in the Cochrane Risk of Bias and the ROBINS-I tools.

Certainty of evidence assessment

We used GRADE to rate the certainty of evidence of synthesised findings in the absence of meta-analysis,[9] which indicates the confidence in a pooled result, in this case, remission rate of type 2 diabetes by each diet. GRADE specifies four levels of certainty of evidence (i.e., high, moderate, low, and very low), derived from five domains: risk of bias of RCTs or NRS, consistency of outcomes, relevance to a research question, precision of effect size, and publication bias.

ESM Results

1) Additional results of an umbrella review of published meta-analyses of diets for weight loss in type 2 diabetes Umbrella review.

Methodological quality of meta-analyses included in the umbrella review of diets for weight loss in type 2 diabetes

Using the conventional quality assessment tools, most of the included meta-analyses were of 'critically low' (n=9)[10-18] to 'low' (n=3)[19-21] quality. Only seven meta-analyses, five for LCDs,[22-26] one for liquid meal replacement,[27] and one for very-low energy diets[28] were assessed as 'high quality'. Detailed assessment for all domains/items is shown in ESM Table 3. All meta-analyses described their research questions/objectives following PICO (item 1), described the included trials in detail (item 8), performed adequate risk of bias assessment (item 9), and reported a conflict-of-interest statement (item 16). Conversely, the criteria that most of the meta-analyses failed to meet were justification of study design for inclusion in their reviews (item 3); providing a list of excluded studies with reasons (item 7); and reporting on the sources of funding for the meta-analysis or included studies (item 10). Unmet critical domains leading to low or critically low quality were protocol registration (item 2), considering risk of bias in pooled results (item 12-13), and investigation of small study effects, such as publication bias (item 15).

Heterogeneity of meta-analyses included in the umbrella review.

Identified sources of heterogeneity of some meta-analyses are presented in ESM Table 7. Major sources include variations in prescriptions of energy restriction (as distinct from diet-type, and the durations of interventions included within a meta-analysis.

Although diet-types were described, not all meta-analyses specified degree of energy restriction. Six reported participants' self-reported energy intakes: three of LCDs and one of high protein diets providing 5.0-8.4 MJ/day (1200-2000kcal), one of formula-meal replacements at 6.3 MJ/day (1500kcal), and one of VLED providing 1.7-2.1 MJ/day (400-500kcal) (Table 1).

The duration of source trials included in some meta-analyses varied. This is important because weight loss is usually most rapid early in treatment, followed by a plateau at 3-6 months and thereafter variable weight loss maintenance. Including very short-term results (e.g., 4-weeks) may be misleading if the full effect on body weight has not yet developed but including 3-6month results is likely to exaggerate the effect on weight loss. Some included very brief diet treatments of just 4-12 weeks, some included trials of 6 months. In some meta-analyses, results were presented as pooled data combining short and longer durations. Some meta-analyses were restricted to longer-term source trials of 12 months or longer. This information is shown in Fig. 2. For most clinical and

service-planning needs, only longer-term data are appropriate as the biological effects of body weight are mainly relevant to long-term health, and short-term changes in body weight have little health impact. Fig. 3 shows the same data as Fig. 2, restricted to the meta-analyses which included source RCTs of 12-months or longer.

Successive meta-analyses of the same diet type are likely to include similar source RCTs, increasing in number over time. Their inclusion/exclusion criteria and reporting frameworks also differ, potentially influencing the synthesis of findings. A detailed analysis of overlaps in source trials is presented in the section below and ESM Table 8-10. Overlapping of source trials did not introduce variance between the meta-analyses of low glycaemic index, high-protein, or Mediterranean diets but there was surprisingly little overlap in source RCTs between the more numerous meta-analyses for LCDs, as a possible source of heterogeneity.

Overlap analysis of source RCTs of weight loss outcome among published meta-analyses of the same diets

Successive meta-analyses of the same diet-type, which include many of the same source publications, appear to produce conflicting conclusions, and attempt to discredit previous ones. That was a large part of the reason we decided to use an umbrella review process. While several meta-analyses do cover a similar range of primary trials, their inclusion/exclusion criteria and reporting frameworks differ, potentially influencing the syntheses of findings. Some used pooled results from source studies with different durations of treatment: including short-term studies which do not include any period of weight loss maintenance tends to exaggerate the effect size. We have previously shown that meta-analyses reporting greatest effect sizes tend to receive a higher number of citations and greater exposure, regardless of quality.[29]

To provide an account of the totality of the available evidence, showing all the previous meta-analyses, and ranking them by methodological quality, we therefore conducted an overlap review of the n=54 source RCTs included in the 10 meta-analyses of low carbohydrate diets, the largest category where inclusion criteria varied most (ESM Table 8). A total of 54 trials were reported at least once, for weight loss outcome. None of these trials featured in all ten meta-analyses included in the umbrella review. Ten unique studies reporting weight as an outcome were reported in 5 or more of the 10 meta-analyses included. The study of Davis 2009 was the most reported, in 9 out of 10 meta-analyses. This overlap analysis does not alter the overall conclusions of this umbrella review. It showed that there was in fact surprisingly little overlap between the meta-analyses for low carbohydrate diets, as a possible source of heterogeneity.

Among the other diet types for which there are >1 meta-analyses; high-protein diets (ESM Table 9), Mediterranean diet (ESM Table 10), and low-glycaemic index diet, we did not observe a relationship between overlaps of source RCTs and the pooled results among meta-analyses included, as the pooled results were quite similar at around <1 to <2kg of WMD. This amount is of no clinical importance, regardless of duration

of intervention. We have not presented overlap analysis tables for the low glycaemic index diet as there are 2 meta-analyses, one with 24 trials (Zafar 2019), and the other one with 3 trials (Ajala 2013), and these 3 trials were all already included in Zafar 2019.

Effect of weight-loss diet interventions on HbA_{1c}

Low-carbohydrate weight-loss diets

LCDs showed marginally greater HbA_{1c} reduction, by -1.1 mmol/mol (-0.1%), than higher carbohydrate diets during 3-36 months (95%CI -1.9 to -0.1; I^2=7%, p=0.38; GRADE moderate certainty of evidence; ESM Table 14) [22]. Results were only different from control for studies at 3-6 months, showing greater HbA_{1c} reduction by -2.2 to -3.3 mmol/mol (-0.2 to -0.3%). There was no effect of LCDs on HbA_{1c} in longer term trials, ≥12 months [22-25]. One meta-analysis reported complete case data, showing the greatest HbA_{1c} reduction by -5.1 mmol/mol (-0.47%) at 6 months following LCDs compared to higher carbohydrate diets [26].

High-protein weight-loss diets

High-protein diet meta-analyses (all critically low quality) had conflicting results for HbA_{1c} reduction (ESM Table 14) [10,12,16]. A recent critically low-quality meta-analysis showed no difference in HbA_{1c} reduction between high- and low-protein diets (WMD -1.1 mmol/mol [-0.1%], 95%CI -3.3 to 1.1 [-0.3% to 0.1%]; n=227, 4 RCTs; I^2=3%, p=0.38) [12]. An older meta-analysis of critically-low quality, found greater HbA_{1c} reduction with high-protein diets (2 RCTs; -3.3 mmol/mol [-0.3%], 95%CI -4.4 to -2.2 [-0.4% to -0.2%]; I^2=60%, p=0.11) [10].

Mediterranean weight-loss diets

Mediterranean diets, in low and critically-low-quality meta-analyses, showed reduction in HbA_{1c} greater than control diets, by -3.3 to -4.4 mmol/mol (-0.3 to -0.4%), but high heterogeneity in the pooled results: I^2 67% to 82% [10,19]. Notably, the control interventions included combinations of no diet (usual care) and various diets including LFD, LCD, or the ADA diet (ESM Table 14). A network meta-analysis (low-quality) found that Mediterranean diets were more effective than LFD on HbA_{1c} reduction (-4.9 mmol/mol [-0.45%], 95%CI -6.0 to -3.7 [-0.55% to -0.34%]; 4 RCTs of direct evidence; p for heterogeneity=0.36) [18].

Formula meal replacements

One high-quality meta-analysis of nine RCTs, 931 participants, reported significantly greater HbA_{1c} reduction with meal replacements than energy-restricted diets (-4.4 mmol/mol [-0.4%], 95%CI -7.7 to -2.2 [-0.7% to -0.2%]; I^2=87%, p<0.001) [27].

Other weight-loss diets

High MUFA diets showed no difference in HbA_{1c} reduction than control diets (critically low quality) [13]. Vegetarian and low glycaemic index diets showed significantly greater reductions in HbA_{1c} than controls (-3.3

mmol/mol [-0.3%], 95%CI -5.5 to -1.1 [-0.5% to -0.1%]; n=369, 7 RCTs; I^2=26%, p=0.23; low-quality meta-analysis; and -1.1 mmol/mol [-0.1%], 95%CI -2.6 to -0.3 [-0.24% to -0.03%]; 3 RCTs; I^2=80%, p=0.007; critically low-quality meta-analysis respectively; ESM Table 14) [10,14].

2) Additional results of a systematic review of diets for type 2 diabetes remission

Methodological quality of included studies in the systematic review of diets for type 2 diabetes remission

Only two RCTs of total diet replacement were conducted with T2D remission as a primary or secondary outcome, both were of low risk of bias.[30-32] The remaining RCTs were ancillary analysis of available original trial data or extended follow-up period to evaluate remission and were rated as having some concerns for risk of bias (Table 2 and ESM Fig. 3).[33-35] A non-randomised controlled study[36] and all single arm intervention studies[37-43] were of serious and critical risk of bias respectively, due to bias in confounding and bias in selection of participants into the study (ESM Table 17).

ESM Table 1: Search strategy

**Umbrella review of published meta-analyses of diets for weight loss**	**Systematic review of diets for type 2 diabetes remission**
Inception to 04 Feb 2020, updated 07 May 2021<br>MEDLINE (OVID)<br>1. Weight Loss/ or weight loss.mp.<br>2. weight management.mp.<br>3. weight reduction.mp.<br>4. Diet/<br>5. diet*.mp. [mp=title, abstract, original title, name of substance word, subject heading word, floating sub-heading word, keyword heading word, organism supplementary concept word, protocol supplementary concept word, rare disease supplementary concept word, unique identifier, synonyms]<br>6. nutrition*.mp. or Nutrition Therapy/<br>7. food.mp. or Food/<br>8. type 2 diabetes.mp. or Diabetes Mellitus, Type 2/<br>9. T2D.mp.<br>10. diabetes.mp.<br>11. type 1 diabetes.mp. or Diabetes Mellitus, Type 1/<br>12. Pregnancy in Diabetics/ or Diabetes, Gestational/ or GDM.mp.<br>13. gestational*.mp.<br>14. Pregnancy/ or pregnan*.mp.<br>15. children.mp. or Child/<br>16. (Grading of Recommendations Assessment, Development and Evaluation).mp. [mp=title, abstract, original title, name of substance word, subject heading word, floating sub-heading word, keyword heading word, organism supplementary concept word, protocol supplementary concept word, rare disease supplementary concept word, unique identifier, synonyms]<br><br>17. grading procedure.mp.<br>18. systematic review.mp. or "Systematic Review"/<br>19. meta-analysis.mp. or Meta-Analysis/<br>20. low-grade.mp.<br>21. high-grade.mp.<br>22. (rat or rats or mouse or mice or rodent* or rabbit* or in vitro or animal model).m_titl.<br>23. 1 or 2 or 3<br>24. 4 or 5 or 6 or 7<br>25. 8 or 9 or 10<br>26. 16 or 17 or 18 or 19<br>27. 11 or 12 or 13 or 14 or 15<br>28. 20 or 21<br>29. 23 and 24 and 25 and 26<br>30. 29 not 27<br>31. 30 not 28<br>32. 31 not 22<br><br>Web of Science Core Collection<br>TOPIC: (Weight loss OR weight management OR weight reduction) AND TOPIC: (diet OR diets OR dietary OR nutrition* OR food) AND TOPIC: (type 2 diabetes OR T2D OR diabetes) AND TOPIC: ("Grading of Recommendations Assessment Development and Evaluation" OR "grading procedure" OR "systematic review" OR meta-analysis OR meta analysis) NOT TOPIC: (type 1 diabetes OR GDM OR gestational* OR pregnan* OR children) NOT TOPIC: (low-grade OR high-grade) NOT TITLE: (rat OR rats OR mouse OR mice OR rodent* OR rabbit* OR "in vitro" OR "animal model")<br><br>PubMed<br>(diet OR diets OR dietary OR nutrition* OR food) AND (weight loss OR weight management OR weight reduction) AND (type 2 diabetes OR T2D OR diabetes) AND ((Grading of Recommendations Assessment, Development and Evaluation) OR (systematic review OR meta-analysis OR meta analysis) OR (grading procedure)) NOT ((type 1 diabetes) OR GDM OR gestational* OR pregnan* OR children) NOT (low-grade OR high-grade) NOT (rat OR rats OR mouse OR mice OR rodent* OR rabbit* OR (in vitro) OR (animal model)	Inception to 04 Aug 2020, updated 10 May 2021<br>Embase (OVID)<br>1. type 2 diabetes.mp. or non-insulin dependent diabetes mellitus/<br>2. remission.ab,ti.<br>3. "diet*".ab,ti.<br>4. intensive lifestyle intervention.ab,ti.<br>5. 3 or 4<br>6. 1 and 2 and 5<br>7. bypass.ab,ti.<br>8. surgery.ab,ti.<br>9. gastrectomy.ab,ti.<br>10. 7 or 8 or 9<br>11. 6 not 10<br><br>PubMed<br>((("intensive lifestyle intervention"[Title/Abstract] OR "diet"[Title/Abstract]) AND ("diabetes mellitus, type 2"[MeSH Terms] OR "diabetes mellitus, type 2"[MeSH Terms] OR "type 2 diabetes mellitus"[All Fields] OR "type 2 diabetes"[All Fields])) AND (("remission"[Title/Abstract]) OR (reversal[Title/Abstract]))) NOT ((bypass[Title/Abstract]) OR (surgery[Title/Abstract]))<br><br>CENTRAL<br>1. MeSH descriptor: [Diabetes Mellitus, Type 2] explode all trees<br>2. (type 2 diabetes):ti,ab,kw<br>3. (remission):ti,ab,kw<br>4. #1 OR #2<br>5. #4 AND #3<br>6. (diet):ti,ab,kw<br>7. (intensive lifestyle intervention):ti,ab,kw<br>8. #6 OR #7<br>9. #8 AND #5<br>10. (bypass):ti,ab,kw<br>11. (surgery):ti,ab,kw<br>12. #10 OR #11<br>13. #9 NOT #12

ESM Table 2: AMSTAR 2 check list items [1]

AMSTAR 2 items	Overall quality rating
**Critical domains**<br>• *Item 2. Did the report of the review contain an explicit statement that the review methods were established prior to the conduct of the review and did the report justify any significant deviations from the protocol?*<br>• *Item 4. Did the review authors use a comprehensive literature search strategy?*<br>• *Item 9. Did the review authors use a satisfactory technique for assessing the risk of bias (RoB) in individual studies that were included in the review?*<br>• *Item 11. If meta-analysis was performed did the review authors use appropriate methods for statistical combination of results?*<br>• *Item 12. If meta-analysis was performed, did the review authors assess the potential impact of RoB in individual studies on the results of the meta-analysis or other evidence synthesis?*<br>• *Item 13. Did the review authors account for RoB in individual studies when interpreting/ discussing the results of the review?*<br>• *Item 14. Did the review authors provide a satisfactory explanation for, and discussion of, any heterogeneity observed in the results of the review?*<br>• *Item 15. If they performed quantitative synthesis did the review authors carry out an adequate investigation of publication bias (small study bias) and discuss its likely impact on the results of the review?*<br>**Non-critical domains**<br>• *Item 1. Did the research questions and inclusion criteria for the review include the components of PICO?*<br>• *Item 3. Did the review authors explain their selection of the study designs for inclusion in the review?*<br>• *Item 5. Did the review authors perform study selection in duplicate?*<br>• *Item 6. Did the review authors perform data extraction in duplicate?*<br>• *Item 7. Did the review authors provide a list of excluded studies and justify the exclusions?*<br>• *Item 8. Did the review authors describe the included studies in adequate detail?*<br>• *Item 10. Did the review authors report on the sources of funding for the studies included in the review?*<br>• *Item 16. Did the review authors report any potential sources of conflict of interest, including any funding they received for conducting the review?*	**Critical domains, where biases could seriously affect the validity of the pooled results, were identified as following:**<br>• protocol registration<br>• comprehensive literature searching<br>• statistical analysis of combined results including investigating the causes of heterogeneity<br>• methods of the risk of bias (RoB) assessment<br>• the impact of RoB on pooled results, discussion and conclusion<br>• assessment of publication bias.<br><br>**We rated the overall quality of the included meta-analyses as following:**<br>High<br>'Yes' for all critical domains, and could have 'No' up to three non-critical domains<br>Moderate<br>'No' for more than three non-critical domains<br>Low<br>'No' for one critical domain<br>Critically low<br>'No' for more than one critical domain

ESM Table 3: Quality assessment and overall rating judgement of published meta-analyses using AMSTAR-2 criteria

Authors	1. Did the research questions and inclusion criteria for the review include the components of PICO?	2. Did the report of the review contain an explicit statement that the review methods were established prior to the conduct of the review and did the report justify any significant deviations from the protocol?	3. Did the review authors explain their selection of the study designs for inclusion in the review?	4. Did the review authors use a comprehensive literature search strategy?	5. Did the review authors perform study selection in duplicate?	6. Did the review authors perform data extraction in duplicate?	7. Did the review authors provide a list of excluded studies and justify the exclusions?	8. Did the review authors describe the included studies in adequate detail?	9. Did the review authors use a satisfactory technique for assessing the risk of bias (RoB) in individual studies that were included in the review?	10. Did the review authors report on the sources of funding for the studies included in the review?	11. If meta-analysis was performed did the review authors use appropriate methods for statistical combination of results?	12. If meta-analysis was performed, did the review authors assess the potential impact of RoB in individual studies on the results of the meta-analysis or other evidence synthesis?	13. Did the review authors account for RoB in individual studies when interpreting/ discussing the results of the review?	14. Did the review authors provide a satisfactory explanation for, and discussion of, any heterogeneity observed in the results of the review?	15. If they performed quantitative synthesis did the review authors carry out an adequate investigation of publication bias (small study bias) and discuss its likely impact on the results of the review?	16. Did the review authors report any potential sources of conflict of interest, including any funding they received for conducting the review?	AMSTAR 2 Quality [a]
Ajala 2013 [10]	Yes	No	No	Partial	No	No	No	Yes	Yes	No	No	No	No	No	No	Yes	Critically low
Fan 2016 [11]	Yes	No	No	Yes	No	Yes	No	Yes	Partial	No	Yes	Yes	Yes	Yes	No	Yes	Critically low
Goldenberg 2021 [26]	Yes	Yes	No	Yes	Yes	Yes	No	Yes	Yes	No	Yes	Yes	Yes	Yes	Yes	Yes	High
Huo 2015 [19]	Yes	No	No	Partial	Yes	Yes	No	Yes	Yes	No	Yes	Yes	Yes	Yes	Yes	Yes	Low
Korsmo-Haugen 2019 [22]	Yes	Yes	No	Yes	Yes	Yes	Yes	Yes	Yes	No	Yes	Yes	Yes	Yes	Yes	Yes	High
McArdle 2019 [21]	Yes	Yes	No	Yes	Yes	Yes	No	Yes	Yes	No	Yes	Yes	Yes	Yes	No	Yes	Low
Meng 2017 [20]	Yes	No	No	Partial	Yes	Yes	No	Partial	Partial	No	Yes	Yes	Yes	Yes	Yes	Yes	Low
Naude 2014 [25]	Yes	Yes	No	Yes	Yes	Yes	Yes	Yes	Yes	No	Yes	Yes	Yes	Yes	Yes	Yes	High
Noronha 2019 [27]	Yes	Yes	Yes	Yes	Yes	Yes	No	Yes	Yes	Yes	Yes	Yes	Yes	Yes	Yes	Yes	High
Pan 2019 [18]	Yes	Yes	No	Yes	Yes	Yes	No	Partial	Yes	No	Yes	No	No	Yes	No	Yes	Critically low
Pfeiffer 2020 [12]	Yes	No	No	No	No	No	Yes	Yes	Yes	No	Yes	Yes	Yes	Yes	Yes	Yes	Critically low
Qian 2016 [13]	Yes	No	No	Yes	No	Yes	No	Yes	Yes	No	Yes	No	No	Yes	Yes	Yes	Critically low
Rehackova 2016 [28]	Yes	Yes	Yes	Yes	Yes	Yes	Yes	Yes	Yes	Yes	Yes	Yes	Yes	Yes	Yes	Yes	High
Sainsbury 2018 [24]	Yes	Yes	No	Yes	Yes	Yes	No	Yes	Yes	No	Yes	Yes	Yes	Yes	Yes	Yes	High
Snorgaard 2017 [17]	Yes	No	No	No	No	Yes	No	Yes	Yes	No	No	No	No	No	No	Yes	Critically low
van Zuuren 2018 [23]	Yes	Yes	No	Yes	Yes	Yes	Yes	Yes	Yes	Yes	Yes	Yes	Yes	Yes	Yes	Yes	High
Viguiliouk 2019 [14]	Yes	Yes	No	Yes	No	Yes	No	Yes	Yes	Yes	Yes	No	No	Yes	Yes	Yes	Critically low
Zafar 2019 [15]	Yes	Yes	No	Yes	Yes	Yes	No	Yes	Yes	Yes	Yes	No	No	Yes	Yes	Yes	Critically low
Zhao 2018 [16]	Yes	No	No	Partial	No	Yes	No	Yes	Yes	No	Yes	No	No	Yes	Yes	Yes	Critically low
Vitale 2020 [44]	Yes	No	Yes	Partial	No	No	Yes	Yes	Yes	No	n.a.	n.a.	Yes	Yes	n.a.	Yes	Low [b]

Authors	1. Did the research questions and inclusion criteria for the review include the components of PICO?	2. Did the report of the review contain an explicit statement that the review methods were established prior to the conduct of the review and did the report justify any significant deviations from the protocol?	3. Did the review authors explain their selection of the study designs for inclusion in the review?	4. Did the review authors use a comprehensive literature search strategy?	5. Did the review authors perform study selection in duplicate?	6. Did the review authors perform data extraction in duplicate?	7. Did the review authors provide a list of excluded studies and justify the exclusions?	8. Did the review authors describe the included studies in adequate detail?	9. Did the review authors use a satisfactory technique for assessing the risk of bias (RoB) in individual studies that were included in the review?	10. Did the review authors report on the sources of funding for the studies included in the review?	11. If meta-analysis was performed did the review authors use appropriate methods for statistical combination of results?	12. If meta-analysis was performed, did the review authors assess the potential impact of RoB in individual studies on the results of the meta-analysis or other evidence synthesis?	13. Did the review authors account for RoB in individual studies when interpreting/ discussing the results of the review?	14. Did the review authors provide a satisfactory explanation for, and discussion of, any heterogeneity observed in the results of the review?	15. If they performed quantitative synthesis did the review authors carry out an adequate investigation of publication bias (small study bias) and discuss its likely impact on the results of the review?	16. Did the review authors report any potential sources of conflict of interest, including any funding they received for conducting the review?	**AMSTAR 2 Quality [a]**
Welton 2020 [45]	Yes	No	No	No	No	No	No	Partial	No	No	n.a.	n.a.	No	Yes	n.a.	Yes	**Critically low [b]**

[a] Criteria for overall quality rating by AMSTAR-2 (see ESM Table 2)

- High - 'Yes' for all critical domains, and could have 'No' up to three non-critical domains
- Moderate - 'No' for more than three non-critical domains
- Low - 'No' for one critical domain
- Critically low - 'No' for more than one critical domain

[b] systematic review without meta-analysis.

ESM Table 4: GRADE assessment on the pooled result of the meta-analyses included in the umbrella review of diets for weight loss in type 2 diabetes.

Authors	Duration	No. of trials	No. of patients	Risk of bias	Inconsistency	Indirectness	Imprecision	Publication bias	GRADE [a]	Downgrade	Reasons
Goldenberg 2021 [26]	6 mo	18	882	Not serious	Not serious	Not serious	Not serious	strongly suspected	Moderate [b]	5	Visual inspection and Egger's plot were suggestive of publication bias (P=0.02)
	12 mo	7	499	Not serious	Not serious	Not serious	Not serious	strongly suspected	Moderate [b]	5	Analysis for publication bias at 12 months was underpowered (k<10), but cautiously rated down for 12-month data as well.
Korsmo-Haugen 2019 [22]	3 mo to 3 yr	17	1587	Serious	Not serious	Not serious	Not serious	Undetected	Moderate [b]	1	Majority of the evidence is from studies at high- or unclear risk of bias
	3-6 mo	7	424	Serious	Not serious	Not serious	Not serious	Undetected	Moderate	1	Majority of the evidence is from studies at high- or unclear risk of bias
	>12 mo	10	1163	Serious	Not serious	Not serious	Not serious	Undetected	Moderate	1	Majority of the evidence is from studies at high- or unclear risk of bias
Van Zuuren 2018 [23]	≤8 wk	5	174	Not serious	Not serious	Not serious	Serious	Undetected	Moderate [b]	4	Low total sample size
	>8-16 wk	4	201	Not serious	Not serious	Not serious	Not serious	Undetected	High [b]	-	The authors of this publication did not downgrade for imprecision. Although the minimal important difference is not established, we consider a reduction of <5% to be not important. Therefore, the effect estimate is rather precise
	>16-26 wk	7	537	Not serious	Serious	Not serious	Serious	Undetected	Low [b]	2,4	I^2=88%. The 95% CI includes both benefit of the low-carbohydrate diet and no difference between the diets. The authors of this publication considered a reduction of 5% to be important (5–10 kg in most studies).
	>26 wk (mean 52wk)	5	483	Not serious	Not serious	Not serious	Not serious	Undetected	High [b]	-	The authors of this publication did not downgrade for imprecision. The 95% CI did not include appreciable harm or benefit. We considered a reduction of 5% to be important (5–10 kg in most studies).
	2yr	2	176	Not serious	Not serious	Not serious	Serious	Undetected	Moderate	4	Low total sample size
Sainsbury 2018 [24]	3 mo	4	321	Serious	Not serious	Not serious	Serious	Undetected	Low	1,4	Majority of the evidence is from studies at high- or unclear risk of bias. Low total sample size
	6 mo	4	274	Serious	Not serious	Not serious	Serious	Undetected	Low	1,4	Majority of the evidence is from studies at high- or unclear risk of bias. Low total sample size
	12 mo	3	281	Serious	Not serious	Not serious	Serious	Undetected	Low	1,4	Majority of the evidence is from studies at high- or unclear risk of bias. Low total sample size
Naude 2014 [25]	3-6 mo	5	599	Serious	Not serious	Not serious	Serious	Undetected	Low [b]	1,4	Majority of the evidence is from studies at high- or unclear risk of bias. Difference in mean weight loss ranges from a loss of 1.25 to a gain of 2.9 kilograms.
	1-2 yr	4	492	Serious	Not serious	Not serious	Serious	Undetected	Low [b]	1,4	Majority of the evidence is from studies at high- or unclear risk of bias. The 95% confidence interval includes both a loss of 2.08 kg and a gain of 3.89 kg.
McArdle 2019 [21]	12-105 wk (median52wk)	7	353	Serious	Not serious	Not serious	Serious	Undetected	Low	1,4	Majority of the evidence is from studies at high- or unclear risk of bias. Low total sample size
	12-104 wk (median26wk)	5	239	Serious	Not serious	Not serious	Serious	Undetected	Low	1, 4	Majority of the evidence is from studies at high- or unclear risk of bias. Low total sample size
Meng 2017 [20]	3-24 mo	8	590	Serious	Not serious	Not serious	Not serious	Undetected	Moderate	1	Included RCTs with varying risk of bias.

Authors	Duration	No. of trials	No. of patients	Risk of bias	Inconsistency	Indirectness	Imprecision	Publication bias	GRADE [a]	Downgrade	Reasons
Snorgaard 2017 [17]	<1 yr	7	741	Not serious	Not serious	Not serious	Not serious	Undetected	High [b]	-	Did not rate down due to no relevant clinical difference (narrow CI).
	≥1 yr	6	771	Not serious	Not serious	Not serious	Not serious	Undetected	High [b]	-	Did not rate down due to no relevant clinical difference (narrow CI).
Fan 2016 [11]	3 mo to 4 yr	10	997	Serious	Serious	Not serious	Not serious	strongly suspected	Very low	1,2,5	Included RCTs with varying risk of bias. I^2=94%, and possible publication bias.
Ajala 2013 [10]	6 mo to 1 yr	9	844	Serious	Serious	Serious	Not serious	Undetected	Very low	1,2,3	Included RCTs with varying risk of bias. No heterogeneity reported. Some source RCTs mixed patients with and without diabetes.
Pfeiffer 2020 [12]	4-15 mo	5	265	Very serious	Not serious	Not serious	Not serious	Undetected	Low [b]	6	Downgraded 2 levels due to high risk of bias RCTs.
Zhao 2018 [16]	4 wk to 2 yr	16	1059	Serious	Not serious	Not serious	Not serious	Undetected	Moderate	1	Included RCTs with varying risk of bias.
Ajala 2013 [10]	1 yr	2	137	Serious	Serious	Not serious	Serious	Undetected	Very low	1,2,4	Included RCTs with varying risk of bias. No heterogeneity reported. Low total sample size
Huo 2015 [19]	4 wk to 2 yr	6	835	Serious	Not serious	Not serious	Not serious	Undetected	Moderate	1	Included RCTs with varying risk of bias.
Ajala 2013 [10]	6 mo to 1 yr	4	1397	Serious	Serious	Serious	Not serious	Undetected	Very low	1,2,3	Included RCTs with varying risk of bias. No heterogeneity reported. Some source RCTs mixed patients with and without diabetes.
Noronha 2019 [27]	12-52 wk	9	931	Not serious	Serious	Not serious	Not serious	Undetected	Moderate [b]	2	I^2=84%
Rehackova 2016 [28]	3 mo	2	100	Serious	Not serious	Not serious	Serious	Undetected	Low	1,4	High risk of bias trials and low total sample size.
	6 mo	2	100	Serious	Not serious	Not serious	Serious	Undetected	Low	1,4	High risk of bias trials and low total sample size.
Qian 2016 [13]	2-52 wk	16	1081	Serious	Not serious	Not serious	Not serious	Undetected	Moderate	1	Included RCTs with varying risk of bias.
Viguiliouk 2019 [14]	4-74 wk	6	532	Not serious	Not serious	Serious	Not serious	Undetected	Moderate [b]	3	Serious indirectness for the effect of vegetarian dietary patterns on body weight as majority of the trials (5/6 trials for body weight) had a follow-up duration <1 year.
Zafar 2019 [15]	2 wk to 12 mo	24	1488	Serious	Not serious	Not serious	Not serious	Undetected	Moderate	1	Included RCTs with varying risk of bias.
Ajala 2013 [10]	6 mo to 1 yr	3	357	Serious	Serious	Not serious	Serious	Undetected	Very low	1,2,4	Included RCTs with varying risk of bias. No heterogeneity reported. Low total sample size

[a] GRADE level for certainty of evidence

- High - we are very confident that the true effect lies close to that of the estimate of the effect.
- Moderate - we are moderately confident in the effect estimate: the true effect is likely to be close to the estimate of the effect, but there is a possibility that it is substantially different.
- Low - our confidence in the effect estimate is limited: the true effect may be substantially different from the estimate of the effect.
- Very low - we have very little confidence in the effect estimate: the true effect is likely to be substantially different from the estimate of effect

[b] GRADE was assessed and quoted by the meta-analysis authors. The present umbrella review authors did not assess GRADE for this meta-analysis.

1. Downgraded by one level due to risk of bias
2. Downgraded by one level due to inconsistency.
3. Downgraded by one level due to indirectness.
4. Downgraded by one level due to imprecision.
5. Downgraded by one level due to possible publication bias.
6. Downgraded by two levels due to very serious risk of bias.

ESM Table 5: List of excluded full texts with reasons

Authors	Title	Reasons	Doi
**Umbrella review of published meta-analyses of diets for weight loss**			
Pfeiffer et al	The Effects of Different Quantities and Qualities of Protein Intake in People with Diabetes Mellitus	already included (duplicate)	10.3390/nu12020365
Zhao et al	High protein diet is of benefit for patients with type 2 diabetes: An updated meta-analysis	already included (duplicate)	10.1097/MD.0000000000013149
Barron et al	[Meal replacement efficacy on long-term weight loss: a systematic review]	duplicate	10.1590/S0212-16112011000600011
Dong et al	Effects of high-protein diets on body weight, glycaemic control, blood lipids and blood pressure in type 2 diabetes: meta-analysis of randomised controlled trials	included trial with exercise co-intervention	10.1017/S0007114513002055
Huntriss et al	The interpretation and effect of a low-carbohydrate diet in the management of type 2 diabetes: a systematic review and meta-analysis of randomised controlled trials	included trial with orlistat (weight loss drug) co-intervention	10.1038/s41430-017-0019-4
Castaneda-Gonzalez et al	Effects of low carbohydrate diets on weight and glycemic control among type 2 diabetes individuals: a systemic review of RCT greater than 12 weeks	no pooled weight loss, and individual studies were already pooled in other SR in the umbrella review	10.1590/S0212-16112011000600013
Kirk et al	Restricted-carbohydrate diets in patients with type 2 diabetes: a meta-analysis	no pooled weight loss, most of RCTs (n=8/9) included was of short duration <8 weeks and small sample size. Only 1 of 9 RCTs was of 26 weeks and already pooled in other more recent SR in the umbrella review.	10.1016/j.jada.2007.10.003
Toumpanakis et al	Effectiveness of plant-based diets in promoting well-being in the management of type 2 diabetes: a systematic review	no pooled weight loss, individual studies in T2D were already pooled in other SR in the umbrella review	10.1136/bmjdrc-2018-000534
Tran et al	Effects of Plant-Based Diets on Weight Status: A Systematic Review	no pooled weight loss, and individual studies in T2D were already pooled in other SR in the umbrella review	10.2147/DMSO.S272802
Barron et al	Meal Replacement Efficacy on Long-Term Weight Loss: A Systematic Review	not English, no pooled weight loss and individual studies in T2D were already pooled in other SR in the umbrella review	10.3305/nh.2011.26.6.5354
Bierbaum et al	Efficacy of diets in the treatment of type 2 diabetes. A systematic review	not English, no pooled weight loss and individual studies in T2D were already pooled in other SR in the umbrella review	10.1007/s11428-014-1323-4
Hernandez Alcantara et al	[Effect of Low Carbohydrate Diets on Weight Loss and Glycosilated Hemoglobin in People with Type 2 Diabetes: Systematic Review]	not English, no pooled weight loss and individual studies in T2D were already pooled in other SR in the umbrella review	10.3305/nh.2015.32.5.9695
Valenzuela Mencia et al	Diets low in carbohydrates for type 2 diabetics. Systematic review	not English, no pooled weight loss and individual studies in T2D were already pooled in other SR in the umbrella review.	10.20960/nh.999
Johannesen et al	Effects of Plant-Based Diets on Outcomes Related to Glucose Metabolism: A Systematic Review	no weight loss outcome	10.2147/DMSO.S265982
Kodama et al	Influence of fat and carbohydrate proportions on the metabolic profile in patients with type 2 diabetes: a meta-analysis	no weight loss outcome	10.2337/dc08-1716
Papamichou et al	Dietary patterns and management of type 2 diabetes: A systematic review of randomised clinical trials	no weight loss outcome	10.1016/j.numecd.2019.02.004
Schwingshackl et al	Effects of monounsaturated fatty acids on glycaemic control in patients with abnormal glucose metabolism: a systematic review and meta-analysis	no weight loss outcome	10.1159/000331214
Garcia-Molina et al	Improving type 2 diabetes mellitus glycaemic control through lifestyle modification implementing diet intervention: a systematic review and meta-analysis	no weight loss outcome	10.1007/s00394-019-02147-6
Martenstyn et al	Impact of weight loss interventions on patient-reported outcomes in overweight and obese adults with type 2 diabetes: a systematic review	no weight loss outcome	10.1007/s10865-020-00140-7
Yu et al	Effects of high-protein diet on glycemic control, insulin resistance and blood pressure in type 2 diabetes: A systematic review and meta-analysis of randomized controlled trials	no weight loss outcome	10.1016/j.clnu.2019.08.008
Silverii et al	Low-carbohydrate diets and type 2 diabetes treatment: a meta-analysis of randomized controlled trials	no weight loss outcome	10.1007/s00592-020-01568-8
Makris and Foster	Dietary approaches to the treatment of obesity	not a systematic review	10.1016/j.psc.2011.08.004

Canuto et al	Nutritional intervention strategies for the management of overweight and obesity in primary health care: A systematic review with meta-analysis	not type 2 diabetes	10.1111/obr.13143
Raben A	Should obese patients be counselled to follow a low-glycaemic index diet? No	not type 2 diabetes	10.1046/j.1467-789x.2002.00080.x
Schwingshackl and Hoffmann	Long-term effects of low glycemic index/load vs. high glycemic index/load diets on parameters of obesity and obesity-associated risks: a systematic review and meta-analysis	not type 2 diabetes	10.1016/j.numecd.2013.04.008
Muscogiuri et al	European Guidelines for Obesity Management in Adults with a Very Low-Calorie Ketogenic Diet: A Systematic Review and Meta-Analysis	not type 2 diabetes	10.1159/000515381
Papadaki et al	The Effect of the Mediterranean Diet on Metabolic Health: A Systematic Review and Meta-Analysis of Controlled Trials in Adults	not type 2 diabetes	10.3390/nu12113342
Choi et al	Impact of a Ketogenic Diet on Metabolic Parameters in Patients with Obesity or Overweight and with or without Type 2 Diabetes: A Meta-Analysis of Randomized Controlled Trials	weight loss presented in mixed group of patients with and without diabetes	10.3390/nu12072005
Leslie et al	Weight losses with low-energy formula diets in obese patients with and without type 2 diabetes: systematic review and meta-analysis	weight loss presented in mixed group of patients with and without diabetes	10.1038/ijo.2016.175
Tobias et al	Effect of low-fat diet interventions versus other diet interventions on long-term weight change in adults: a systematic review and meta-analysis	weight loss presented in mixed group of patients with and without diabetes	10.1016/S2213-8587(15)00367-8
Ross et al	Exploring the highs and lows of very low carbohydrate high fat diets on weight loss and diabetes- and cardiovascular disease-related risk markers: A systematic review	weight loss presented in mixed group of patients with and without diabetes	10.1111/1747-0080.12649
Huang et al	Efficacy of Intermittent or Continuous Very Low-Energy Diets in Overweight and Obese Individuals with Type 2 Diabetes Mellitus: A Systematic Review and Meta-Analyses	weight loss presented in mixed intervention diets, included non-RCT	10.1155/2020/4851671
Alarim et al	Effects of the Ketogenic Diet on Glycemic Control in Diabetic Patients: Meta-Analysis of Clinical Trials	weight loss presented in mixed intervention diets	10.7759/cureus.10796
Borgundvaag et al	Metabolic Impact of Intermittent Fasting in Patients With Type 2 Diabetes Mellitus: A Systematic Review and Meta-analysis of Interventional Studies	weight loss presented in mixed intervention diets; defined very low energy diets as intermittent fasting	10.1210/clinem/dgaa926
Yan et al	Effects of fasting intervention regulating anthropometric and metabolic parameters in subjects with overweight or obesity: a systematic review and meta-analysis	weight loss presented in mixed intervention diets; included non-RCT	10.1039/d0fo00287a
Kloecker et al	Efficacy of low- and very-low-energy diets in people with type 2 diabetes mellitus: A systematic review and meta-analysis of interventional studies	weight loss presented as change from baseline in relation to level of energy deficit, not diet	10.1111/dom.13727
Yuan et al	Effect of the ketogenic diet on glycemic control, insulin resistance, and lipid metabolism in patients with T2DM: a systematic review and meta-analysis	weight loss presented as change from baseline	10.1038/s41387-020-00142-z
Sellahewa et al	A Systematic Review of Evidence on the Use of Very Low Calorie Diets in People with Diabetes	cannot get full text.<br>no pooled weight loss. Another SR of this diet was already included in the umbrella review.	10.2174/1573399812666151005123431
**Systematic review of diets for type 2 diabetes remission**			
Aung, T. et al.	Low calorie liquid diet (LCD) for weight reduction and remission of Type 2 diabetes: Single-centre group pilot project	meeting abstract	10.1111/dme.31_13571
Hung, J. D. et al.	Impact of a low carbohydrate diet on traditional CVD risk factors in people with features of the metabolic syndrome and type 2 diabetes	meeting abstract	10.1177/2047487318786171
Pesta, D. et al.	Targeting Remission of Type 2 Diabetes Using a Digital Education and Behavior Change Program Improves Insulin Sensitivity and Liver Fat Content in Type 2 Diabetes	meeting abstract	10.2337/db19-1915-P
Tucker, S. et al.	Effect of Macronutrients on Metabolic Parameters and Remission of Type 2 Diabetes	meeting abstract	10.1136/jim-2020-SRM.544
Sakr, M. et al.	Diabetes remission after nonsurgical intensive lifestyle intervention in obese patients with type 2 diabetes	meeting abstract of included study (duplicate)	10.2337/db15-1929-2253
Yakubovich, N. et al.	Remission evaluation of metabolic interventions in type 2 diabetes (REMIT) - results of a randomized controlled pilot trial	meeting abstract of McInnes et al. (duplicate)	10.1016/j.jcjd.2014.07.137
Dambha-Miller, H. et al.	Behaviour change, weight loss and remission of Type 2 diabetes: a community-based prospective cohort study	no report T2D remission for each intervention arm/control	10.1111/dme.14122
Kempf, K. et al.	Individualized Meal Replacement Therapy Improves Clinically Relevant Long-Term Glycemic Control in Poorly Controlled Type 2 Diabetes Patients	no report T2D remission	10.3390/nu10081022

Moriconi E. et al.	Very-Low-Calorie Ketogenic Diet as a Safe and Valuable Tool for Long-Term Glycemic Management in Patients with Obesity and Type 2 Diabetes	Unclear remission data, reported only those who discontinued medication but did not mention blood glucose threshold	10.3390/nu13030758
Saslow L. R. et al	A randomized pilot trial of a moderate carbohydrate diet compared to a very low carbohydrate diet in overweight or obese individuals with type 2 diabetes mellitus or prediabetes	Unclear remission data, whether discontinue diabetes medication	10.1371/journal.pone.0091027
Tay J. et al	A very low-carbohydrate, low-saturated fat diet for type 2 diabetes management: a randomized trial	Unclear remission data	10.2337/dc14-0845
Zaharia, O. P. et al	Improving insulin sensitivity, liver steatosis and fibrosis in type 2 diabetes by a food-based digital education-assisted lifestyle intervention program: a feasibility study	Unclear remission data, whether discontinue diabetes medication	10.1007/s00394-021-02521-3
Goldenberg et al	Efficacy and safety of low and very low carbohydrate diets for type 2 diabetes remission: systematic review and meta-analysis of published and unpublished randomized trial data	Unclear remission data of source RCTs; a systematic review	10.1136/bmj.m4743
McInnes, N. et al.	Piloting a Remission Strategy in Type 2 Diabetes: Results of a Randomized Controlled Trial	drug intervention	10.1210/jc.2016-3373
Ried-Larsen, M. et al.	Type 2 diabetes remission 1 year after an intensive lifestyle intervention: A secondary analysis of a randomized clinical trial	exercise intervention	10.1111/dom.13802
Lean, M. E. J. et al.	Baseline Predictors and Influence of Early Weight Loss during an Intensive Weight Management Programme on Remission of Type 2 Diabetes after 12 Months-Post-Hoc Analysis of the Diabetes Remission Clinical Trial (DiRECT)	Post hoc analysis of DiRECT + meeting abstract	10.2337/db18-291-OR
Melhem S. et al	Effect of Weight Loss by Low-Calorie Diet on Cardiovascular Health in Type 2 Diabetes: An Interventional Cohort Study	Secondary analysis of previously included study	10.3390/nu13051465
Webster, C. et al.	Diet, Diabetes Status, and Personal Experiences of Individuals with Type 2 diabetes Who Self-Selected and Followed a Low Carbohydrate High Fat diet	observational self-reported dieters	10.2147/DMSO.S227090
Stentz, F.	Pathobiology of Remission of Type 2 Diabetes	trial registry	https://clinicaltrials.gov/show/NCT03832725

SR, systematic review; T2D, type 2 diabetes

ESM Table 6: Protocols, data sources and search used in meta-analyses in the umbrella review.

Author	Protocol	Databases	Date search	Limits
**Goldenberg 2021 [26]**	Yes	5 DB: CENTRAL, Medline, Embase, CINAHL, CAB, and grey literature<br>3 trial registries (for example, clinicaltrials.gov) and four additional grey literature sources (for example, BIOSIS Citation Index, ProQuest Dissertations & Theses Global)	Inception to 25 August 2020	None
**Korsmo-Haugen 2019 [22]**	Yes	6 DB: Medline, Embase, CENTRAL, CINAHL, Food Science Source and SweMed+	Inception to January 2016	RCTs between 1983 and January 2016<br>Included English, Danish, Norwegian and Swedish.
**van Zuuren 2018 [23]**	Yes	11 DB: Medline, PubMed, Embase, Web of Science, Cochrane Library, CENTRAL, Emcare, Academic Search Premier, ScienceDirect, Latin American and Caribbean Health Science Information database, and Índice Bibliográfico Español en Ciencias de Salud.<br>+ 5 trials registries	Inception to 21 March 2017	None
**Sainsbury 2018**	Yes	5 DB: Medline, Embase, CINAHL, Global Health, and CENTRAL	1 January 1980 to 31 August 2016	None
**Naude 2014 [25]**	Yes.	3 DB: Medline, Embase and CENTRAL	Inception to 19 March 2014	English and human
**McArdle 2019 [21]**	Yes	5 DB: Medline, EMBASE, CINAHL, Cochrane Library, and DARE, and grey literature	between 1976 and April 2018	1976 onwards (because of the introduction of HbA_{1c} measurement at this time)
**Meng 2017 [20]**	NR	3 DB: Medline, Embase, and the Cochrane Library	Inception to January 2017.	No publication time and language restriction
**Snorgaard 2017 [17]**	NR	3 DB: Embase, Medline, and the Cochrane Library	Inception to October 2014	Published between January 2004 to October 2014<br>English and Scandinavian
**Fan 2016 [11]**	NR	4 DB: Embase, PubMed, Medline, and the Cochrane Library	Inception to May 2014.	no language restriction
**Pfeiffer 2020 [12]**	NR	1 DB: PubMed	NR	Filter clinical trials, species: human<br>English
**Zhao 2018 [16]**	NR	2 DB: PubMed and Embase databases	Inception to June 2018	None
**Zafar 2019 [15]**	Yes	3 DB: PubMed, Cochrane Library, and EMBASE<br>3 trials registries: clinicaltrials.gov, and the WHO clinical trials databases	Inception to 1 March 2019	None
**Huo 2015 [19]**	NR	3 DB: PubMed, Cochrane Library and EMBASE	Inception to February 2014	None
**Noronha 2019 [27]**	Yes	3 DB: Medline, Embase, and CENTRAL	Inception to 10 December 2018	None
**Rehackova 2016 [28]**	Yes	12 DB: all EBM Reviews (1991), CAB Abstracts (1973), CINAHL (1994), Embase (1980), HMIC (1979), Ovid MEDLINE(R) In-Process & Other Non-Indexed Citations and Ovid MEDLINE(R) (1946), and PsychINFO (1806). Hand-searched PubMed (1984), Web of Knowledge (1983), The Cochrane library and CRD	Inception to February 2014.	None.<br>Included English, French, Polish, Czech, German, Hungarian, Dutch, and Japanese.
**Qian 2016 [13]**	NR	3 DB: PubMed, Medline, and CENTRAL	Inception to 31 March 2015	Limit to RCTs published in English
**Viguiliouk 2019 [14]**	Yes	3 DB: Medline, Embase, and CNETRAL	Inception to 26 February 2018	None
**Ajala 2013 [10]**	NR	3 DB: PubMed, Embase, and Google Scholar	Inception to July 2011	None
**Pan 2019 [18]**	Yes	3 DB: PubMed, Embase and CENTRAL	Inception to May 2017	None

NR, not reported; DB, databases; CENTRAL, Cochrane Central Register of Controlled Trials; RCT, randomised controlled trial.

ESM Table 7: Methods about investigation of sources of heterogeneity in the meta-analyses that showed heterogeneity of their pooled results of weight loss outcome

Author, year	Meta-analysis model	Investigation of sources of heterogeneity if present	Source of heterogeneity
Geldenberg 2021	Random effect model	Subgroup analysis by risk of bias: low risk vs. high risk/some concern	Heterogeneity for weight loss outcome at 6 months (I^2=66%, P<0.001) was explained by risk of bias; lower risk of bias shows larger effect with I^2=0%.
van Zuuren 2018	Random effect model	Sensitivity analysis to examine the effect of excluding studies at overall high risk of bias and the impact of excluding studies that were the cause of substantial heterogeneity.	Caused by 2 studies that showed the greatest differences in body weight favouring the LCD group.<br>1. LCD group being presumably more adherent due to the counselling ahead of the study.<br>2. Another study, LCD had far fewer energy (2.5-3.4 MJ [600–800 kcal] in the “active” phase) than the LFD (2.1-4.2 MJ [500–1000 kcal] restriction according to each individual’s basal metabolic rate).
McArdle 2019	Random effect model	Subgroup analysis by level of carbohydrate intake<br>- Moderate carbohydrate 130-225g<br>- Low carbohydrate <130g<br>- Very low carbohydrate <50g<br>Did not conduct meta-regression due to less than 10 RCTs for each carbohydrate level.	High heterogeneity remained in very low carbohydrate subgroup.<br><br>No further investigation was reported.
Fan 2016	Random effect model	Sensitivity analysis by exclusion of any single study.	Sensitivity analysis by exclusion of any single study did not materially alter the overall result.<br><br>No further investigation was reported.
Noronha 2019	Random effect model	Sensitivity analysis by removing each individual trial.<br><br>Sensitivity analyses were also conducted based on study duration and type of liquid meal replacement.<br><br>Studies whose removal explained the heterogeneity, changed the significance of the effect, or altered the effect size by 10% or more were considered influential.<br><br>If 10 or more trials were available per outcome, then potential sources of heterogeneity were also explored through a priori subgroup analyses using meta-regression by baseline values, study design, follow up, type of liquid meal replacement, comparator arm, risk of bias, and diabetes duration.	Due to <10 studies included the authors did not conduct subgroup analyses as planned.<br><br>There was presence of unexplained heterogeneity.
Rehackova 2016	Random effect model	Plot between weight loss difference and energy prescription difference of included studies.	Greater difference in energy prescription between intervention diets and control diets was correlated with greater difference in weight loss between the two diets.<br><br>Authors did not comment on source of heterogeneity regarding weight loss outcome.

LCD, low-carbohydrate diets; LFD, Low-fat diets

ESM Table 8: Overlap analysis of trials of low carbohydrate diets reported in the ten meta-analyses included in the umbrella review

Source RCTs	Meta-analyses										Count
	Goldenberg 2021	Korsmo-Haugen 2019	McArdle 2019	van Zuuren 2018	Sainsbury 2018	Meng 2017	Snorgaard 2017	Fan 2016	Naude 2014	Ajala 2013	
Davis 2009/2012	x	x	x	x	x	x	x	x		x	9
Guldbrand 2012	x	x		x	x	x	x	x	x		8
Yamada 2014	x	x	x	x	x	x	x	x			8
Elhayani 2010		x	x	x	x		x	x		x	7
Daly 2006	x	x	x		x	x		x			6
Tay 2014/2015	x		x	x	x	x	x				6
Westman 2008	x	x	x		x	x				x	6
Wolever 2008		x	x	x	x		x			x	6
Kreb 2012		x	x		x		x		x		5
Larsen 2011		x	x		x		x		x		5
Goldstein 2011	x	x	x			x					4
Saslow 2014	x				x	x	x				4
Brinkworth 2004		x			x				x		3
Iqbal 2010	x		x				x			x	3
Parker 2002			x		x				x		3
Brehm 2009			x		x						2
de Bont 1981			x	x							2
Esposito 2009			x					x			2
Jonasson 2014		x	x								2
Jönsson 2009	x		x								2
Luger 2013		x			x						2
Nielsen 2005				x				x			2
Pedersen 2014		x			x						2
Samaha 2003								x		x	2
Saslow 2017	x		x								2
Sato 2017	x		x								2
Stern 2004								x		x	2
Walker 1995			x	x							2
Watson 2016			x		x						2
Ben-Avraham 2009			x								1
Bozzetto 2012				x							1
Brunerova 2007					x						1
Dyson 2007			x								1
Dyson 2010	x										1
Facchini 2003		x									1

Source RCTs	Meta-analyses										Count
	Goldenberg 2021	Korsmo-Haugen 2019	McArdle 2019	van Zuuren 2018	Sainsbury 2018	Meng 2017	Snorgaard 2017	Fan 2016	Naude 2014	Ajala 2013	
Goday 2016				x							1
Gumbiner 1998				x							1
Haimoto 2008										x	1
Hockaday 1978				x							1
Jenkins 2014		x									1
Lerman-Garber 1995				x							1
McLaughlin 2007		x									1
Milne 1994			x								1
Miyashita 2004				x							1
Morris 2019	x										1
Nisak 2013			x								1
Nishimori 2018	x										1
Nutall 2012				x							1
Perna 2019	x										1
Strychar 2009					x						1
Vlachos 2011	x										1
Wycherley 2010					x						1
Yancy 2010										x	1
Zadeh 2018	x										1
**Pooled results of meta-analysis, WMD (95%CI)**	18	17	25	16	20	8	10	9	5	9	
6 months (<12 months)	-3.5 (-5.3, -1.7)	-0.2 (-0.3, -0.1)	-	-0.3 (-0.5, -0.02)	-0.36 (-0.6, -0.1)	-	-0.3 (-0.1, -0.1)	-	0.2 (-0.0, 0.4)	-	
12 months or longer	0.3 (-1.0, 1.6)	0 (-0.1, 0.1)	-	-0.4 (-0.6, -0.1)	-0.17 (-0.4, 0.1)	-	0 (-0.04, 0.13)	-	0.01 (-0.3, 0.3)	-	
Combining all duration	-	-0.4 (-0.9, 0.2)	SMD -0.13 (-0.34, 0.08)	-	-	-0.4 (-0.6, -0.3)	-	SMD -0.5 (-0.9, -0.2)	-	-0.1 (-0.2, -0)	

Cells with green colour were source RCTs that their relevant meta-analysis defined as 'moderate' carbohydrate diets (26-45%E carbohydrate) and some of these RCTs were also featured in other meta-analyses as a LCD (<45%E carbohydrate).

ESM Table 9: Overlap analysis of trials of high protein diets reported in the three meta-analyses included in the umbrella review

Source RCTs	Meta-analyses			Count
	Pfeifer 2020	Zhao 2018	Ajala 2013	
Brinkworth 2004	x	x	x	3
Larsen 2011	x		x	2
Luger 2013	x	x		2
Wycherley 2010	x	x		2
Pedersen 2013	x			1
Cheryl L 2014		x		1
David RJ 2013		x		1
Guldbrand 2012		x		1
Khoo J 2012		x		1
Krebs 2012		x		1
Daly 2006		x		1
Gannon 2004		x		1
Nerylee 2016		x		1
Papakonstantinuo 2010		x		1
Parker 2002		x		1
Sargrad 2005		x		1
Tay 2014		x		1
Westman 2008		x		1
**Pooled results of meta-analysis, WMD (95%CI)**	5	16	2	
Combining all duration	-1.21 (-2.17, -0.24)	-0.09 (-0.21, 0.04)	0.4 (-1,1.8)	

ESM Table 10: Overlap analysis of trials of Mediterranean diets reported in the two meta-analyses included in the umbrella review

Source RCTs	Meta-analyses		Count
	Huo 2015	Ajala 2013	
Toobert 2003	x	x	2
Esposito 2009	x	x	2
Elhayany 2010	x	x	2
Rodriguez-Villar 2004	x		1
Karantonis 2006	x		1
Brehm 2009	x		1
Salas-Salvado´ 2008		x	1
**Pooled results of meta-analysis, WMD (95%CI)**	6	4	
Combining all duration	-0.29 (-0.55, -0.04)	-1.84 (-2.54, -1.15)	

ESM Table 11: Dietary patterns/regimens vs. control diets on weight loss (kg) stratified by overall confidence using AMSTAR-2 quality assessment

AMSTAR quality	Authors	Intervention diet	Control diet	Energy restriction	Duration	No. of trials (n)	No. of participants	WMD in weight loss between diets (kg)	95%CI	I-square (%) [a]	GRADE [b]	Note
**Low-carbohydrate diets**												
High	Goldenberg 2021 [26]	LCD <26%E or <130g CHO	Any diets >26%E CHO	RCTs with E restriction & ad libitum	6 mo	18	882	-3.5	-5.3 to -1.7	63%, p<0.001	Moderate [c]	5
		results from complete case data			12 mo	7	499	0.3	-1.0 to 1.6	NR	Moderate [c]	5
High	Korsmo-Haugen 2019 [22]	LCD <40%E CHO	>40%E CHO	RCTs with E restriction & ad libitum	3 mo to 3 yr	17	1587	-0.4	-0.9 to 0.2	29%, p=0.12	Moderate [c]	1
					3-6 mo	7	424	-0.9	-1.9 to 0.2	33%, p=0.18	Moderate	1
					>12 mo	10	1163	0.1	-0.3 to 0.6	0%, p=0.59	Moderate	1
High	Van Zuuren 2018 [23]	LCD <40%E	LFD <30%E	RCTs with E restriction & ad libitum	≤8 wk	5	174	-0.81	-2.1 to 0.5	12%, p=0.33	Moderate [c]	4
					>8-16 wk	4	201	-2.04	-3.2 to 0.9	0%, p=0.47	High [c]	-
					>16-26 wk	7	537	-2.51	-5.4 to 0.4	88%, p<0.001	Low [c]	2,4
					>26 wk (mean 52 wk)	5	483	-0.19	-1.7 to 1.3	0%, p=0.72	High [c]	-
					2yr	2	176	-0.14	-1.6 to 1.4	0%, p=0.75	Moderate	4
High	Sainsbury 2018 [24]	LCD <130g CHO	>45%E CHO	RCTs with E restriction & ad libitum	3 mo	4	321	-2.47	-3.3 to -1.6	0%, p=0.66	Low	1,4
					6 mo	4	274	-1.07	-2.5 to 0.4	33%, p=0.21	Low	1,4
					12 mo	3	281	0.58	-0.8 to 2.0	0%, p=0.52	Low	1,4
High	Naude 2014 [25]	<40%E CHO 5.3-8.6 MJ (1260-2054 kcal)	45–65%E CHO & isoenergetic to LCD arm. 5.9-7.5 MJ (1416-1800 kcal)	RCTs with E restriction and non-restriction. Excluded ad libitum.	3-6 mo	5	599	0.82	-1.3 to 2.9	0%, p=0.93	Low [c]	1,4
					1-2 yr	4	492	0.91	-2.1 to 3.9	33%, p=0.21	Low [c]	1,4
Low	McArdle 2019 [21]	Very LCD <50g CHO	>50%E CHO	NR	12-105 wk (median 52wk)	7	353	SMD -0.01	-0.7 to 0.7	89%, p<0001	Low	1,4
		LCD <130g CHO			12-104 wk (median 26wk)	5	239	SMD -0.43	-0.7 to -0.1	24%, p=0.25	Low	1, 4
Low	Meng 2017 [20]	LCD <130g or 26%E	Normal or hi-CHO diet	NR	3-24 mo	8	590	-0.94	-1.9 to 0.1	36%, p=0.14	Moderate	1
Critically low	Snorgaard 2017 [17]	LCD <45%E CHO, either hi protein or hi-fat	Hi-CHO 45-50%E CHO	NR	<1 yr	7	741	0	-1.0 to 1.0	NR	High [c]	-
					≥1 yr	6	771	0.2	-1.0 to 1.4	NR	High [c]	-

Critically low	Fan 2016 [11]	LCD <130 g/d	LFD, hi-CHO, conventional diet, ADA diet.	RCTs with E restriction & ad libitum	3 mo to 4 yr	10	997	SMD -0.82	-1.4 to -0.3	94%, p<0.001	Very low	1,2,5
Critically low	Ajala 2013 [10]	LCD	LFD, low-GI, Mediterranean, conventional hi-CHO diet	NR	6 mo to 1 yr	9	844	-0.69	-1.8 to 0.4	NR	Very low	1,2,3
**Hi-protein diets**												
Critically low	Pfeiffer 2020 [12]	Hi-protein diet >20%E protein, in exchange for CHO. 5.1-8.5 MJ (1219-2029 kcal)	Lower protein intake (<20%E) 5.2-7.5 MJ (1235-1785 kcal)	E restricted	4-15 mo	5	265	-1.2	-2.2 to -0.2	5%, p=0.38	Low [c]	6
Critically low	Zhao 2018 [16]	Hi-protein diets	Not specified. 40-60% CHO 10-20% protein 10-42% fat	NR	4 wk to 2 yr	16	1059	SMD -0.1	-0.2 to 0.04	0%, p=0.65	Moderate	1
Critically low	Ajala 2013 [10]	Hi-protein diets	low-protein, hi-CHO diets	NR	1 yr	2	137	0.4	-1.0 to 1.8	NR	Very low	1,2,4
**Mediterranean diets**												
Low	Huo 2015 [19]	Mediterranean style diets	usual diet, usual care, ADA diet, LFD, LCD	NR	4 wk to 2 yr	6	835	-0.3	-0.6 to -0.04	0%, p=0.92	Moderate	1
Critically low	Ajala 2013 [10]	Mediterranean diets	usual care, ADA diets	NR	6 mo to 1 yr	4	1397	-1.8	-2.5 to -1.2	NR	Very low	1,2,3
**Formula meal replacement**												
High	Noronha 2019 [27]	Liquid meal replacement that replaced one to 3 main meals	Low-energy diets using food-exchange systems (4/9 trials), self-selected low-energy foods (4/9 trials), and a diet book (1/9 trials)	E restricted, Average 6.3 MJ or 1500 kcal (5.0-6.9 MJ or 1,195–1,659 kcal) both arms	12-52 wk	9	931	-2.4	-3.3 to -1.4	84%, p<0.001	Moderate [c]	2
**Very-low energy diet**												
High	Rehackova 2016 [28]	Very low energy diets 1.7-2.1 MJ/d (400-500 kcal) 8-12 wk duration	Low energy diet 4.2-6.3 MJ/d (1000-1500 kcal)	E restricted	3 mo	2	100	-6.6	-9.5 to -3.7	58%, p=0.12	Low	1,4
					6 mo	2	100	-5.7	-11.1 to -0.4	58%, p=0.12	Low	1,4
**Hi MUFA diets**												
Critically low	Qian 2016 [13]	Hi-MUFA diet No specified criteria	Hi-CHO diet	NR	2-52 wk	16	1081	-1.6	-2.9 to -0.2	0%, p=1	Moderate	1
**Vegetarian diet**												
Critically low	Viguiliouk 2019 [14]	Vegetarian diet including vegan to lacto-ovo-vegetarian	LFD, conventional diabetes diet, usual diet	8 RCTs E-restricted 1 RCT E-balance	4-74 wk	6	532	-2.2	-3.0 to -1.3	21%, p=0.28	Moderate [c]	3
**Low glycaemic index diets**												

Critically low	Zafar 2019 [15]	Low-glycaemic index diet	hi-GI, LFD, LCD, and weight loss diets	Some of included trials aimed for E restriction	2 wk to 12 mo	24	1488	-1.15	-2.43 to 0.13	0%, p=0.64	Moderate	1
Critically low	Ajala 2013 [10]	Low glycaemic index diet	Hi-fibre, h-GI, ADA diets	NR	6 mo to 1 yr	3	357	1.4	-1.6 to 4.4	NR	Very low	1,2,4

E, energy; LCD, low-carbohydrate diet; CHO, carbohydrate; LFD, low-fat diet; GI, glycaemic index; ADA, American Diabetes Association; MUFA, mono-unsaturated fatty acids; RCT, randomised controlled trial; WMD, weighted mean difference; SMD, standardised mean difference; NR, not reported; NA, Not Appropriate; mo, month; wk, week; yr, year.

[a] I-square >50% and P<0.10 is considered evidence of substantial heterogeneity.

[b] GRADE level for certainty of evidence, appropriate when AMSTAR quality is Moderate or High [46]

- High - we are very confident that the true effect lies close to that of the estimate of the effect.
- Moderate - we are moderately confident in the effect estimate: the true effect is likely to be close to the estimate of the effect, but there is a possibility that it is substantially different.
- Low - our confidence in the effect estimate is limited: the true effect may be substantially different from the estimate of the effect.
- Very low - we have very little confidence in the effect estimate: the true effect is likely to be substantially different from the estimate of effect

[c] GRADE was assessed and quoted by the meta-analysis authors. The present umbrella review authors did not assess GRADE for this meta-analysis.

1. Downgraded by one level due to risk of bias
2. Downgraded by one level due to inconsistency.
3. Downgraded by one level due to indirectness.
4. Downgraded by one level due to imprecision.
5. Downgraded by one level due to possible publication bias.
6. Downgraded by two levels due to very serious risk of bias.

ESM Table 12: A network meta-analysis by Pan 2019[18] including 10 RCTs of varying dietary regimens

**Weight loss**	**LFD**	**LCD**	**hi-CHO**
**Mediterranean diets**	-1.18 (-1.99, -0.37) [a]	-1.28 (-3.75, 1.18) [b]	-0.24 (-5.97, 5.49) [c]
**LFD**		-0.11 (-2.43, 2.22) [c]	0.93 (-4.85, 6.72) [b]
**LCD**			1.04 (-5.19, 7.28) [b]
**HbA_{1c}**	**LFD**	**LCD**	**hi-CHO**
**Mediterranean diets**	-0.45 (-0.55, -0.34) [a]	-0.15 (-1.23, 0.94) [b]	-0.1 (-0.51, 0.31) [c]
**LFD**		0.3 (-0.78, 1.38) [c]	0.35 (-0.07, 0.77) [b]
**LCD**			0.05 (-1.12, 1.21) [b]

LFD, low-fat diet; LCD, low-carbohydrate diet; CHO, carbohydrate.
Minus value indicates that diets in the column on the left achieved greater weight loss than diets in the row.
[a] Direct evidence: 4 RCTs
[b] Indirect evidence: 0 RCT
[c] Direct evidence: 2 RCTs

ESM Table 13: Systematic reviews (without meta-analysis) of intermittent fasting in T2D

	Vitale 2020 [44]	Welton 2020 [45]
**AMSTAR-2 quality**	Low quality	Critically low quality
**Protocol**	NR	NR
**Data source**	PubMed, CINAHL, and MEDLINE	Medline and Embase
**Search**	1 January 2000 to 21 February 2020.	1 January 2000 to 1 July 2020
**Design of studies included**	3 RCTs (n=54-137) involving T2D<br>- 2 RCTs for IER<br>- 1 RCT for TRF	3 RCTs (n=54-137) involving T2D
**Cochrane RoB**	High RoB	Did not assess
**Diet types**	**1) IER**: 5:2 regimen = 2.1-2.5 MJ/day (500–600 kcal) for 2 days + ad libitum intake for 5 days. Duration 12-52 wk<br>**2) TRF**: Two meals/day (6–10 a.m. and 12– 4 p.m.) Duration 12 wk<br>50-55% CHO, 20-25% protein, <30% fat	Same to Vitale
**Control diet**	**CER**: 5.0-6.3 MJ/day (1200–1500 kcal) Duration 12-52 wk<br>45% CHO, 30% protein, 25% fat	Same to Vitale
**Weight loss**		
**IER** [47,48]	Similar weight loss between IER and control (p>0.05)<br>12wk: ~6kg (6%) from baseline both arms<br>52wk: 4kg from baseline both arms	Same to Vitale
**TRF** [49]	12wk<br>TRF: -3.7kg from baseline vs<br>CER: -2.3kg from baseline<br>Between group P< 0.001	Same to Vitale
**HbA_{1c}**	No difference in HbA_{1c} reduction between IER or TRF and CER	Same to Vitale
**Systematic review Authors' conclusion**	The majority of the studies demonstrated insignificant differences between intermittent fasting and continuous energy restriction for measures of glycated haemoglobin a1c and body composition. More data on intermittent fasting in adults with obesity and type 2 diabetes are needed to determine its benefits within this patient population	Intermittent fasting shows promise for the treatment of obesity. To date, the studies have been small and of short duration. Longer-term research is needed
**GRADE**	Very low certainty [1,2,3]	Very low certainty [1,2,3]

RoB, risk of bias; IER, intermittent energy restriction; TRF, time-restricted feeding; CER, continuous energy restriction; NR, not reported; RCT, randomised controlled trial.
1. Downgraded by one level due to risk of bias.
2. Downgraded by one level due to imprecision (small sample size)
3. Downgraded by one level due to publication bias (reporting bias)

ESM Table 14: Dietary patterns/regimens vs. control diets on HbA_{1c} stratified by overall confidence using AMSTAR-2 quality assessment

AMSTAR quality	Authors	Intervention diet	Control diet	Duration	No. of trials	No. of participants	WMD in HbA_{1c} between diets	95%CI	I-square (%) [a]	GRADE [b]	Note
**Low-carbohydrate diets**											
High	Goldenberg 2021	LCD <26%E or <130g CHO	Any diets >26%E CHO	6 mo	17	747	-0.47%	-0.6 to -0.3	NR	High [c]	-
	Results from complete case data						-5.1 mmol/mol	-6.6 to -3.3			
				12 mo	8	489	-0.23%	-0.5 to 0	NR	Moderate [c]	7
							-2.5 mmol/mol	-5.5 to 0			
High	Korsmo-Haugen 2019	LCD <40%E CHO	>40%E CHO	3 mo to 3 yr	16	1425	-0.1%	-0.17 to -0.01	7%, p=0.38	Moderate [c]	1
							-1.1 mmol/mol	-1.9 to -0.1			
				3-6 mo	6	395	-0.2%	-0.3 to -0.1	0%, p=0.84	Moderate	1
							-2.2 mmol/mol	-3.3 to -1.1			
				>12 mo	10	1030	0%	-0.1 to 0.1	0%, p=0.55	Moderate	1
							0 mmol/mol	-1.1 to 1.1			
High	Van Zuuren 2018	LCD <40%E	LFD <30%E	≤8 wk	2	42	-1.4%	-2.6 to -0.1	68%, p=0.08	Very low [c]	1,2,4
							-15.3 mmol/mol	-28.4 to -1.1			
				>8-16 wk	4	201	-0.6%	-0.9 to -0.2	54%, p=0.09	Low [c]	1,4
							6.6 mmol/mol	-9.8 to -2.2			
				>16-26 wk	7	539	-0.3%	-0.50 to -0.02	59%, p=0.02	Moderate [c]	4
							-3.3 mmol/mol	-5.5 to -0.2			
				>26 wk (mean 52 wk)	4	390	-0.4%	-0.6 to -0.1	0%, p=0.93	Low [c]	1,4
							-4.4 mmol/mol	-6.6 to -1.1			
				2yr	3	199	0%	-0.4 to 0.4	13%, p=0.32	NR [c]	-
							0 mmol/mol	-4.4 to 4.4			
High	Sainsbury 2018 [24]	LCD <130g CHO	>45%E CHO	3 mo	4	321	-0.47%	-0.7 to -0.2	0%, p=0.68	Very low	1,4,5
							-5.1 mmol/mol	-7.7 to -2.2			
				6 mo	5	328	-0.36%	-0.6 to -0.1	0%, p=0.50	Low	1,4
							-3.9 mmol/mol	-6.6 to -1.1			
				12 mo	3	335	-0.17%	-0.4 to 0.1	0%, p=0.49	Low	1,4
							-1.9 mmol/mol	-4.4 to 1.1			

High	Naude 2014	<40%E CHO	45–65%E CHO & isoenergetic to LCD arm	3-6 mo	5	599	0.2%	-0.0 to 0.4	0%, p=0.88	Moderate [c]	1
							2.2 mmol/mol	-0 to 4.4			
				1-2 yr	4	492	0.01%	-0.3 to 0.3	0%, p=1.00	Moderate [c]	1
							0.1 mmol/mol	-3.3 to 3.3			
Low	McArdle 2019 [21]	Very LCD <50g CHO	>50%E CHO	12-105 wk (median 52wk)	8	467	SMD -0.13	-0.34 to 0.08	19%, p=0.28	Moderate	1
		LCD <130g CHO		≤6 mo	5	239	SMD -0.49	-0.75 to -0.23	0%, p=0.56	Low	1, 4
Low	Meng 2017	LCD <130g or 26%E	Normal or hi-CHO	3-24 mo	9	734	-0.4%	-0.61 to -0.26	19.6%, p=0.26	Low	1,4
							-4.4 mmol/mol	-6.7 to -2.8			
Critically low	Snorgaard 2017	LCD <45%E CHO, either hi protein or hi fat	Hi-CHO diet 45-50%E CHO	3-6 mo	8	809	-0.34%	-0.63 to -0.06	74%, p=0.0003	Moderate	2
							-3.7 mmol/mol	-6.9 to -0.7			
				≥1yr	7	839	0.04%	-0.04 to 0.13	0%, p=0.68	High	-
							0.4 mmol/mol	-0.4 to 1.4			
Critically low	Fan 2016	LCD <130g	LFD, high CHO, conventional diet, ADA diet.	3 mo to 4 yr	11	1141	SMD -0.5	-0.9 to -0.2	88%, p<0.001	Very low	1,2,5
Critically low	Ajala 2013	LCD	LFD, low-GI, hi-CHO, Mediterranean	6 mo to 1 yr	8	799	-0.12%	-0.24 to 0	75%, p=0.002	Very low	1-4
							-1.3 mmol/mol	-2.6 to 0			
**Hi-protein diets**											
Critically low	Pfeiffer 2020	Hi-protein diet >20%E protein, in exchange for CHO	Lower protein intake (<20%E)	3-12 mo	4	227	-0.1%	-0.3 to 0.1	3%, p=0.38	Low [e]	6
							-1.1 mmol/mol	-3.3 to 1.1			
Critically low	Zhao 2018	Hi-protein diets	Not specified	>4 wk	13	933	SMD -0.1	-0.2 to 0.1	0%, p=0.52	Very low	1,4,5
Critically low	Ajala 2013	Hi-protein diets	low-prot, hi-CHO	1 yr	2	137	-0.3%	-0.4 to -0.2	60% p=0.11	Very low	1,2,4
							-3.3 mmol/mol	-4.4 to -2.2			
**Mediterranean diets**											
Low	Huo 2015	Mediterranean style diets	usual diet, usual care, ADA diet, LFD, LCD	4 wk to 2 yr	9	1178	-0.3%	-0.5 to -0.1	67%, p=0.001	Very low	1,2,4,5
							-3.3 mmol/mol	-5.5 to -1.1			
Critically low	Ajala 2013	Mediterranean diets	usual care, ADA diets	6 mo to 1 yr	3	578	-0.4%	-0.6 to -0.2	82%, p=0.004	Very low	1-4
							-4.4 mmol/mol	-6.6 to -2.2			
**Liquid meal replacement**											
High	Noronha 2019	Liquid meal replacement that replaced one to 3 main meals	Low-energy weight loss diets. Total E is isocaloric to intervention diet.	12-52 wk	9	931	-0.4%	-0.7 to -0.2	87%, p<0.001	Low [c]	2,4
							-4.4 mmol/mol	-7.7 to -2.2			

**Hi MUFA diets**											
Critically low	Qian 2016	Hi-MUFA diet<br>No specified criteria	Hi-CHO	2-52 wk	14	925	-0.1%<br>-1.1 mmol/mol	-0.2 to 0.02<br>-2.2 to 0.2	40%, p=0.04	Very low	1,2,4
**Vegetarian diet**											
Critically low	Viguiliouk 2019	Vegetarian diet including vegan to lacto-ovo-vegetarian	LFD, conventional diabetes diet, usual diet	4-74 wk	7	369	-0.3%<br>-3.3 mmol/mol	-0.5 to -0.1<br>-5.5 to -1.1	26%, p=0.23	Moderate [c]	4
**Low glycaemic index diets**											
Critically low	Zafar 2019	Low-glycaemic index diet	high-GI, low-fat, low-carbohydrate, conventional weight loss diets	2 wk to 12 mo	21	1352	SMD -0.2	-0.3 to -0.1	NR	Very low	1,2,4
Critically low	Ajala 2013	Low glycaemic index diet	high fibre, high GI, ADA diets	6 mo to 1 yr	3	357	-0.1%<br>-1.1 mmol/mol	-0.24 to -0.03<br>-2.6 to -0.3	80%, p=0.007	Very low	1,2,4

E, energy; LCD, low-carbohydrate diet; CHO, carbohydrate; LFD, low-fat diet; GI, glycaemic index; ADA, American Diabetes Association; MUFA, mono-unsaturated fatty acids; RCT, randomised controlled trial; WMD, weighted mean difference; SMD, standardised mean difference; NR, not reported; NA, Not Appropriate; mo, month; wk, week; yr, year.

[a] I-square >50% and P<0.10 is considered evidence of substantial heterogeneity.

[b] GRADE level for certainty of evidence, appropriate when AMSTAR quality is Moderate or High [46]

- High - we are very confident that the true effect lies close to that of the estimate of the effect.
- Moderate - we are moderately confident in the effect estimate: the true effect is likely to be close to the estimate of the effect, but there is a possibility that it is substantially different.
- Low - our confidence in the effect estimate is limited: the true effect may be substantially different from the estimate of the effect.
- Very low - we have very little confidence in the effect estimate: the true effect is likely to be substantially different from the estimate of effect

[c] GRADE was assessed and quoted by the meta-analysis authors. The present umbrella review authors did not assess GRADE for this meta-analysis.

1. Downgraded by one level due to risk of bias.
2. Downgraded by one level due to inconsistency.
3. Downgraded by one level due to indirectness.
4. Downgraded by one level due to imprecision, because 95% CI overlaps with the minimally important difference for clinical benefit (-3.3 mmol/mol or -0.3% HbA_{1c}).
5. Downgraded by one level due to publication bias.
6. Downgraded by two levels due to very serious risk of bias.
7. Rated down for imprecision because 95%CI includes small effect, no effect, and small worsening

ESM Table 15: Characteristics of controlled clinical trials reporting T2D remission

Author	Design	A priori outcomes of trial	Analysis	Sample	Duration of T2D	Diet Intervention	Comparator	T2D remission definition	
								Glucose level criteria	Medication criteria
**Formula diets**									
**Lean 2018** DiRECT, UK [30]	RCT	Co-1ry: 15kg weight loss and T2D remission	N/A	ILI (n=149) usual care (n=149)<br><br>Mainly White 98-99%	T2D <6y (mean 3y)	**TDR**<br>3.5-3.6 MJ/d (825-853 kcal) for 12wk then food reintroduction for 2-8wk	Usual care	HbA_{1c} <48 mmol/mol (<6.5%)	At least 2mo off all antidiabetic medications. Stopped all drugs before commencing the trial
**Taheri 2020** DIADEM, Qatar [32]	RCT	1ry: weight loss at 12mo.<br><br>2nd: T2D remission	N/A	TDR (n=70) usual care (n=77)<br><br>Middle East, North Africa	T2D <3y	**TDR**<br>3.3-3.4 MJ/d (800-820 kcal) for 12wk then food reintroduction for 12wk	Usual care	HbA_{1c} <48 mmol/mol (<6.5%)	no pharmacological therapy for diabetes for at least 3 months.<br>Stopped all drugs before commencing the trial
**Gregg 2012** Look AHEAD, USA [34]	RCT	1ry: effect of weight loss on cardiovascular disease incidence	Ancillary observational analysis	ILI (n=2570) DSE (n=2575)<br><br>62% white<br>16% African<br>14% Hispanic	T2D median 5y (IQR 8)	**Liquid meal replacement** provided to assist dietary goals: 2 meal replacements during 0-20wk and then 1 meal replacement thereafter.<br>Goal 5.0-7.5 MJ/d (1200-1800 kcal)	Diabetes support and education	<u>Complete remission:</u><br>FPG <5.6mmol/L; and HbA_{1c} <39 mmol/mol (<5.7%)<br><br><u>Partial remission:</u><br>FPG 5.6-7 mmol/L; and HbA_{1c} 39-48 mmol/mol (5.7%-6.5%)	No antidiabetic medications
**Food based diets**									
**Gutierrez-Mariscal 2021** CARDIOPREV, Spain [35,50]	RCT	1ry: composite incidence of cardiovascular events in established CHD patients	Analysis in a subset cohort of CHD with T2D in CARDIOPREV study (n=183/1002)	MD (n=80) LFD (n=103)	CHD and short duration T2D (screened after inclusion to RCT)	**MD**<br><35%E fat (22% MUFA, 6% PUFA, <10% SFA), 15% proteins, <50%E CHO<br><br>No energy restriction<br><br>Dietary counselling: meal plan, recipes, shopping list.<br><br>Extra virgin olive oil 1 L weekly provided.	**LFD**<br><30% Fat (12–14% MUFA, 6–8% PUFA, <10% SFA), 15% protein, >55% complex CHO<br><br>No energy restriction<br><br>Dietary counselling: meal plan, recipes, shopping list.<br>Food pack provided.	HbA_{1c} <48 mmol/mol (<6.5%), FPG <7 mmol/L,<br>2 h-PG of 75g OGTT < 11.1 mmol/L	no diabetes drug and maintaining these levels for at least two consecutive years.

**Esposito 2014**<br>Italy [33]	RCT	1^{ry}: time to introduction of diabetes medications<br><br>2nd: weight, coronary risk factors	extended postcore RCT follow up	MD (n=108)<br>LFD (n=107)	Newly diagnosed T2D, never used OHA or insulin.	**LCMD**<br>rich in vegetables and whole grains, low red meat - replaced with poultry and fish.<br><50%E CHO, >30%E Fat - main source of added fat 30–50 g of olive oil.<br><br>Restricted energy to women 6.3 MJ/d (1500 kcal)<br>men 7.5 MJ/d (1800 kcal)<br><br>Dietary advice<br>Neither food provided nor paid for	**LFD**<br>rich in whole grains and restricted additional fats, sweets, and high-fat snacks, with the goal <30%E Fat, <10%E SFA<br><br>Restricted energy to women 6.3 MJ/d (1500 kcal)<br>men 7.5 MJ (1800 kcal)<br><br>Dietary advice<br>Neither food provided nor paid for	<u>Complete remission:</u><br>FPG <5.6mmol/L; and HbA_{1c} <39 mmol/mol (<5.7%)<br><br><u>Partial remission:</u><br>FPG 5.6-7 mmol/L; and HbA_{1c} 39-48 mmol/mol (5.7%-6.5%)	Drug naïve patients.
**Mollentze 2019**<br>South Africa [51]	pilot RCT	1^{ry}: T2D remission	pilot	Low energy diet (n=9),<br>standard medical nutrition (n=9)	Insulin treated T2D men with obesity<br><br>T2D ³4y with insulin use >12mo	commercially available low-fat energy-restricted diet primarily consisting of vegetables supplemented with a vegetable soup-based meal plan<br><br>Subjects were booked into a holiday lodge for the first 9 days.<br><br>Not clear whether all food provided or paid for	Energy-restricted diet meal plan to lose weight 0.5-1 kg/wk.<br><br>Dietary advice<br>Neither food provided nor paid for	<u>Complete remission:</u><br>FPG <5.6 mmol/L and HbA_{1c} ≤48 mmol/mol (≤6.5%)<br><br><u>Partial remission:</u><br>FPG ≥5.6 and ≤6.9 mmol/L and HbA_{1c} ≤48 mmol/mol (≤6.5%)	<u>Complete remission:</u> without taking any OHA including insulin.<br><br><u>Partial remission:</u> on metformin only.<br><br>At the time of assessment, patients with remission discontinued OHA.
**Hallberg 2018, Athinarayanan 2019**<br>VIRTA, USA [36,52]	Non-RCT	1^{ry}: HbA1c, Weight loss, medication.<br><br>2nd: T2D remission (mentioned in a 2-y paper)	N/A	CCI (n=262)<br>Usual care (n=87)<br><br>5% African American	T2D mean 8.3y (SD 7.2)	**VLCKD**<br>as part of CCI.<br>CHO <30g/day to achieve nutritional ketosis, protein 1.5g/kg/day, 3–5 servings of non-starchy vegetables, multivitamin, vitamin D3, and Omega-3.<br><br>No energy restriction advised<br><br>Dietary advice<br>Neither food provided nor paid for	Usual care from their own medical providers and diabetes education Program<br><br>Dietary advice from local dietitian not involving in a study<br><br>Neither food provided nor paid for<br><br>Not clear whether energy restriction advised	<u>Complete remission:</u><br>HbA_{1c} <39 mmol/mol (<5.7%)<br><br><u>Partial remission:</u> HbA_{1c} 39-48 mmol/mol (5.7-6.5%)	No glucose-lowering medications, at least 1y.

1^{ry}, primary; 2nd, secondary; mo, month; y, year; T2D, type 2 diabetes; RCT, randomised controlled trial; OHA, oral hypoglycaemic agents; ILI, intensive lifestyle intervention; TDR, total diet replacement; DSE, diabetes support and education; CCI, continuous care intervention; MD, Mediterranean diet; LFD, low-fat diet; CHD, coronary heart diseases; MUFA, mono-unsaturated fatty acids; PUFA, poly-unsaturated fatty acids; SFA, saturated fatty acids; CHO, carbohydrate; LCMD, low-carbohydrate Mediterranean diet; VLCKD, very low-carbohydrate ketogenic diet; FPG, fasting plasma glucose.

ESM Table 16: Characteristics of single arm trials (non-randomised studies) reporting T2D remission

Author	Analysis	Diet intervention	Recommendation post intervention	Assessment time points	Study Population			T2D remission definition	
					Samples	Dropout	Duration of T2D	Glucose level criteria	Medication criteria
**Bynoe 2019**<br>Barbados [37]	Feasibility trial	**TDR** liquid diets<br>3.2 MJ/d (760 kcal)<br>8 wk.	food re-introduction over 4 wk, then solid food until f/u	8wk and 8mo.	Total n=25<br><br>88% Black Caribbean	dropout n=1 at 8mo.	T2D ≤6y	FPG <7 mmol/L	Stopped all drugs before commencing the trial
**Steven 2016**<br>UK [41]	Mechanistic study: glucose metabolism	**TDR** liquid diets<br>2.6-2.9 MJ/d<br>(624-700 kcal)<br>8 wk.	food re-introduction over 2 wk, then solid food until f/u	8-10wk and 8mo	Total n=30<br><br>Ethnicity NR	dropout n=1 at 1wk.<br>not meet weight loss target.	T2D<br>mean 7.3y<br>range 0.5-23y	FPG <7 mmol/L	Stopped all drugs before commencing the trial
**Umphonsathien 2019**<br>Thailand [43]	Efficacy and safety evaluation	**VLED** food based<br>2.5 MJ/d (600 kcal)<br>8 wk.	transition to 6.3 MJ/d (1500 kcal) over 4 wk.	8wk and 12wk	Total n=20<br>Asian	Withdraw consent n=1	T2D<br>median 2y<br>(IQR 0.4-8)	HbA_{1c} <48 mmol/mol (<6.5%),<br>FPG <7 mmol/L	No medication
**Thomas & Shamanna 2018**<br>India [42]	N/A	**VLED** food based<br>2.9 MJ/d (700 kcal)<br>1wk.	dietary advice for ideal body weight until f/u	1y	Total n=9<br>Asian	Dropout n=1 due to lost f/u	T2D<br>median 2.5y<br>(IQR 1.5-6)	HbA_{1c} <48 mmol/mol (<6.5%)	No medication
**Mottalib 2015**<br>Why WAIT, USA [39]	Ancillary observation analysis: only in those who completed 1-y f/u	**Liquid meal replacement**<br>for breakfast & lunch to target 5.0-7.5 MJ/d<br>(1200-1800 kcal)<br>40% CHO, 30% Fat, 30% Protein<br>12 wk.	option to use breakfast and lunch menus, to continue meal replacement, or to use them interchangeably.	1y	Total n=126<br><br>Ethnicity NR	Completed n=88<br><br>Dropout n=36 (30%) at 1y	Not reported	<u>Complete remission:</u><br>FPG <5.6mmol/L; and HbA_{1c} <39 mmol/mol (<5.7%)<br><u>Partial remission:</u><br>FPG 5.6-7 mmol/L; and HbA_{1c} 39-48 mmol/mol (5.7%-6.5%)	No medication
**Dave 2019**<br>India [38]	cohort in clinical practice	**ADA diet**<br>part of lifestyle intervention	continue diet through f/u time points	1y and 5y	Total n=45<br>Asian	dropout n=4 (9%) at 5y	T2D<br>mean 1.9y<br>(SD 2.7)	<u>Complete remission:</u><br>FPG <5.6mmol/L; and HbA_{1c} <39 mmol/mol (<5.7%)<br><u>Partial remission:</u><br>FPG 5.6-7 mmol/L; and HbA_{1c} 39-48 mmol/mol (5.7%-6.5%)	No medication
**Sarathi 2017**<br>India [40]	cohort in clinical practice	**LFD**<br>6.3 MJ/d (1500 kcal), part of lifestyle intervention.<br>60% CHO, 15% Protein, 25% Fat.	continue diet through f/u time points	3mo, 1y and 2y	Total n=32<br>Asian young adults mean age 25y	NA	Newly diagnosed	<u>Complete remission:</u><br>FPG <5.6mmol/L; and HbA_{1c} <39 mmol/mol (<5.7%)<br><u>Partial remission:</u><br>FPG 5.6-7 mmol/L; and HbA_{1c} 39-48 mmol/mol (5.7%-6.5%)	Not mentioned clearly in methods, but in the table showing no medication at the time of assessment

T2D, type 2 diabetes; VLED, very-low energy diet; TDR, total diet replacement; FPG, fasting plasma glucose; NR, not reported; f/u, follow up; wk, week; mo, month; y, year

ESM Table 17: Risk Of Bias In Non-randomised Studies of Interventions (ROBINS-I)

Studies	Diets	Bias due to confounding	Bias in selection of participants into the study	Bias in classification of interventions	Bias due to deviations from intended interventions	Bias due to missing data	Bias in measurement of outcomes	Bias in selection of the reported result	Overall risk of bias [a]
Bynoe 2019	TDR	critical	serious	low	low	low	low	low	**critical**
Steven 2016	TDR	critical	serious	low	low	low	low	moderate	**critical**
Mottalib 2015	Formula meal replacement	critical	serious	low	low	serious	low	moderate	**critical**
Umphonsathien 2019	VLED	critical	serious	low	low	low	low	low	**critical**
Thomas & Shamanna 2018	VLED	critical	serious	low	no information	low	low	serious	**critical**
Dave 2019	ADA	critical	serious	low	low	low	low	moderate	**critical**
Sarathi 2017	Low energy diet	critical	serious	low	low	low	low	moderate	**critical**
Hallberg 2018	Very low-carbohydrate ketogenic diet	serious	serious	low	low	moderate	low	moderate	**serious**

TDR, total diet replacement; VLED, very low-energy diet; ADA, American Diabetes Association.

[a] Overall risk of bias judgement

- Low: low risk of bias for all domains.
- Moderate: low or moderate risk of bias for all domains.
- Serious: serious risk of bias in at least one domain, but not at critical risk of bias in any domain.
- Critical: critical risk of bias in at least one domain.

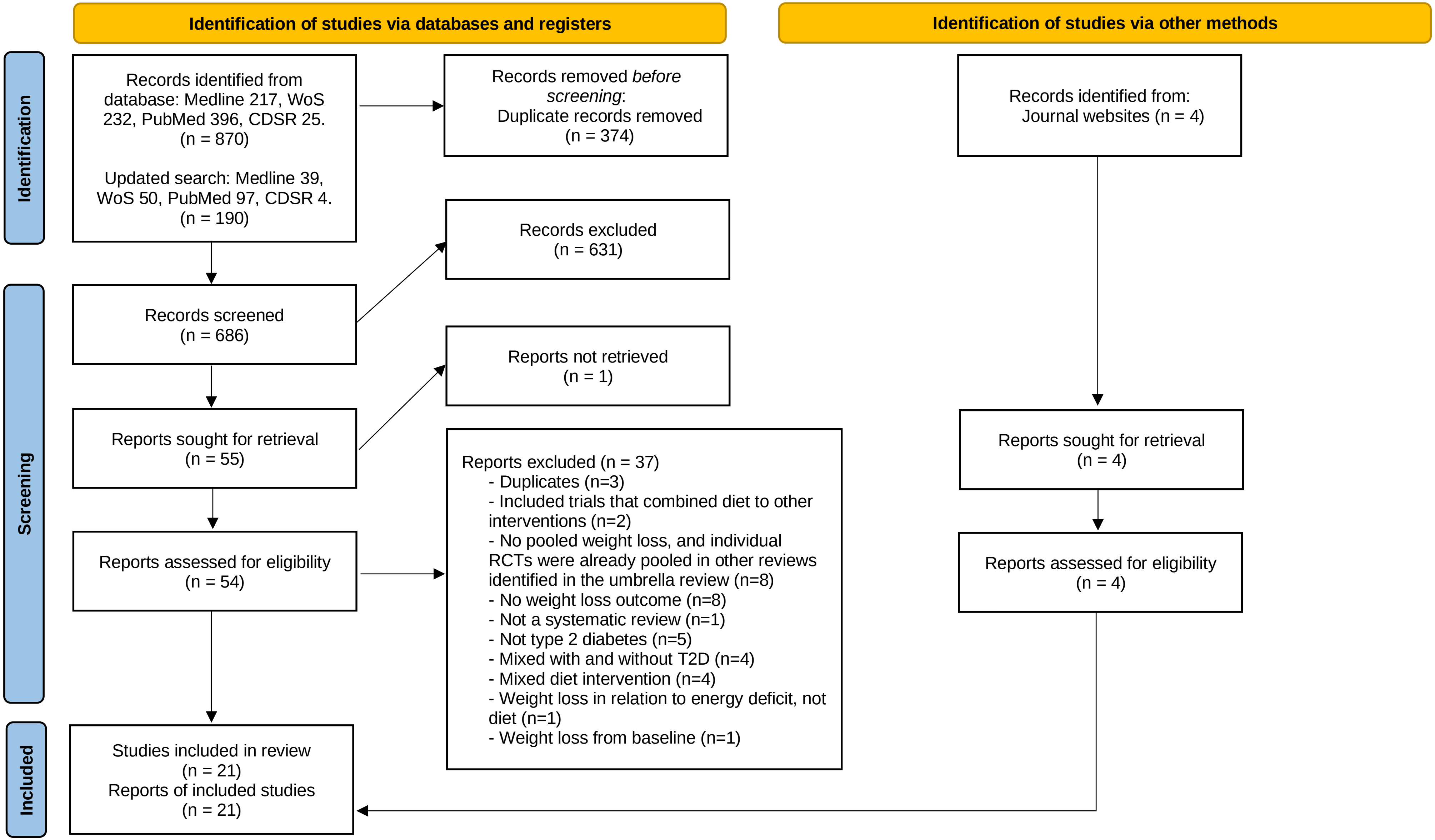

ESM Fig. 1: PRISMA 2020 flow diagram of study selection process of an umbrella review of published meta-analyses for dietary weight management (updated search 7 May 2021)

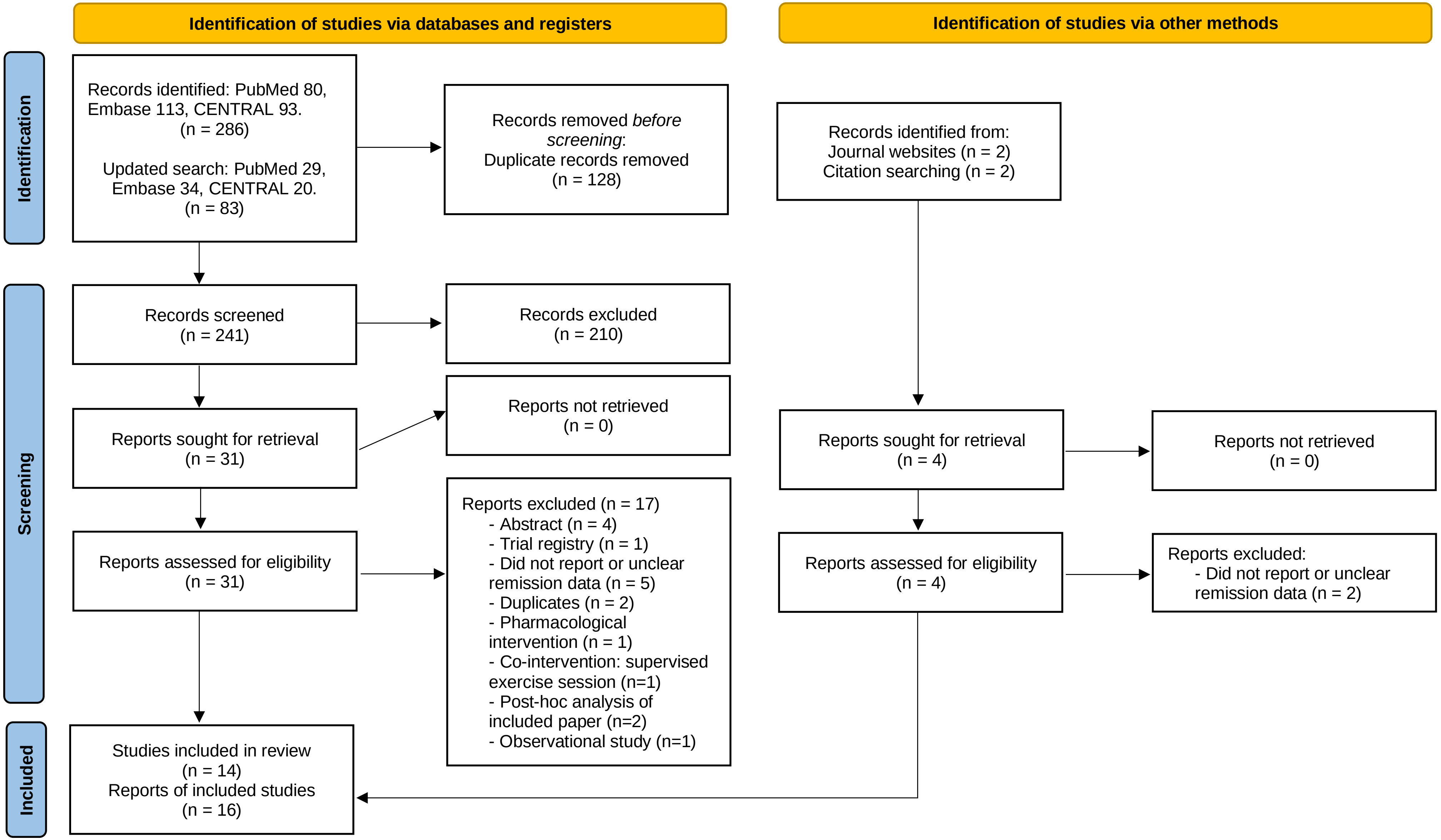

ESM Fig. 2: PRISMA 2020 flow diagram of study selection process of a systematic review of diets for type 2 diabetes remission (updated search 10 May 2021)

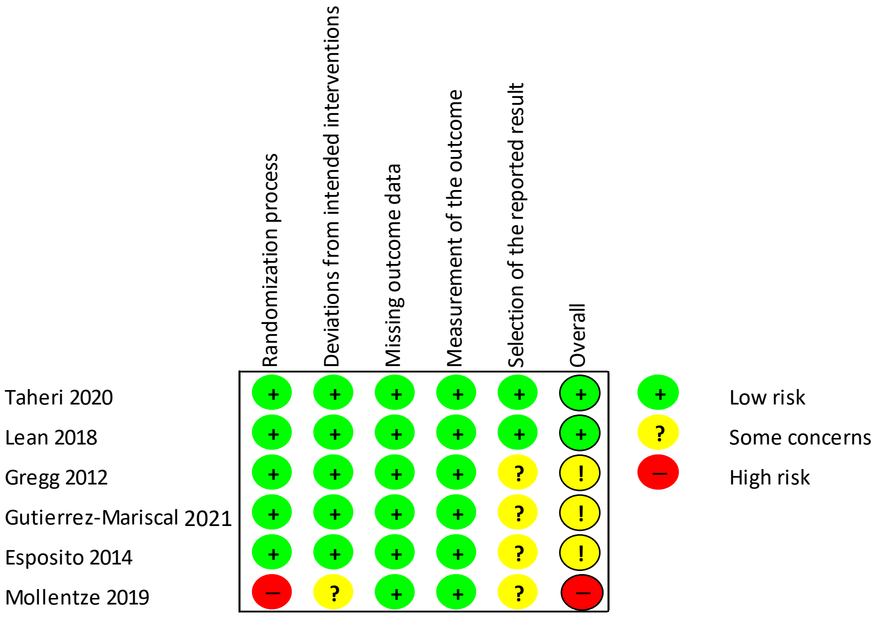

ESM Fig. 3: Cochrane risk of bias assessment 2.0 of randomised controlled trials in a systematic review of diets for type 2 diabetes remission.

References

1. Shea BJ, Reeves BC, Wells G, et al. AMSTAR 2: a critical appraisal tool for systematic reviews that include randomised or non-randomised studies of healthcare interventions, or both. *BMJ* 2017; **358**: j4008.
2. Pieper D, Koensgen N, Breuing J, Ge L, Wegewitz U. How is AMSTAR applied by authors - a call for better reporting. *BMC Med Res Methodol* 2018; **18**(1): 56.
3. Guyatt G, Oxman AD, Akl EA, et al. GRADE guidelines: 1. Introduction-GRADE evidence profiles and summary of findings tables. *J Clin Epidemiol* 2011; **64**(4): 383-94.
4. Guyatt GH, Oxman AD, Kunz R, et al. GRADE guidelines 6. Rating the quality of evidence—imprecision. *Journal of Clinical Epidemiology* 2011; **64**(12): 1283-93.
5. Schunemann HJ, Tugwell P, Reeves BC, et al. Non-randomized studies as a source of complementary, sequential or replacement evidence for randomized controlled trials in systematic reviews on the effects of interventions. *Res Synth Methods* 2013; **4**(1): 49-62.
6. Reeves BC, Deeks JJ, Higgins JPT, Shea B, Tugwell P, Wells GA. Chapter 24: Including non-randomized studies on intervention effects. In: Higgins JPT, Thomas J, Chandler J, et al., editors. Cochrane Handbook for Systematic Reviews of Interventions version 61 (updated September 2020) Cochrane; 2020.
7. Sterne JAC, Savovic J, Page MJ, et al. RoB 2: a revised tool for assessing risk of bias in randomised trials. *BMJ* 2019; **366**: l4898.
8. Sterne JA, Hernan MA, Reeves BC, et al. ROBINS-I: a tool for assessing risk of bias in non-randomised studies of interventions. *BMJ* 2016; **355**: i4919.
9. Murad MH, Mustafa RA, Schunemann HJ, Sultan S, Santesso N. Rating the certainty in evidence in the absence of a single estimate of effect. *Evid Based Med* 2017; **22**(3): 85-7.
10. Ajala O, English P, Pinkney J. Systematic review and meta-analysis of different dietary approaches to the management of type 2 diabetes. *Am J Clin Nutr* 2013; **97**(3): 505-16.
11. Fan YF, Di HJ, Chen GF, Mao XD, Liu C. Effects of low carbohydrate diets in individuals with type 2 diabetes: systematic review and meta-analysis. *Int J Clin Exp Med* 2016; **9**(6): 11166-74.
12. Pfeiffer AFH, Pedersen E, Schwab U, et al. The Effects of Different Quantities and Qualities of Protein Intake in People with Diabetes Mellitus. *Nutrients* 2020; **12**(2).
13. Qian F, Korat AA, Malik V, Hu FB. Metabolic Effects of Monounsaturated Fatty Acid-Enriched Diets Compared With Carbohydrate or Polyunsaturated Fatty Acid-Enriched Diets in Patients With Type 2 Diabetes: A Systematic Review and Meta-analysis of Randomized Controlled Trials. *Diabetes Care* 2016; **39**(8): 1448-57.
14. Viguiliouk E, Kendall CW, Kahleova H, et al. Effect of vegetarian dietary patterns on cardiometabolic risk factors in diabetes: A systematic review and meta-analysis of randomized controlled trials. *Clin Nutr* 2019; **38**(3): 1133-45.
15. Zafar MI, Mills KE, Zheng J, et al. Low-glycemic index diets as an intervention for diabetes: a systematic review and meta-analysis. *Am J Clin Nutr* 2019; **110**(4): 891-902.
16. Zhao WT, Luo Y, Zhang Y, Zhou Y, Zhao TT. High protein diet is of benefit for patients with type 2 diabetes: An updated meta-analysis. *Medicine (Baltimore)* 2018; **97**(46): e13149.
17. Snorgaard O, Poulsen GM, Andersen HK, Astrup A. Systematic review and meta-analysis of dietary carbohydrate restriction in patients with type 2 diabetes. *BMJ Open Diabetes Res Care* 2017; **5**(1): e000354.
18. Pan B, Wu Y, Yang Q, et al. The impact of major dietary patterns on glycemic control, cardiovascular risk factors, and weight loss in patients with type 2 diabetes: A network meta-analysis. *J Evid Based Med* 2019; **12**(1): 29-39.
19. Huo R, Du T, Xu Y, et al. Effects of Mediterranean-style diet on glycemic control, weight loss and cardiovascular risk factors among type 2 diabetes individuals: a meta-analysis. *Eur J Clin Nutr* 2015; **69**(11): 1200-8.
20. Meng Y, Bai H, Wang S, Li Z, Wang Q, Chen L. Efficacy of low carbohydrate diet for type 2 diabetes mellitus management: A systematic review and meta-analysis of randomized controlled trials. *Diabetes Res Clin Pract* 2017; **131**: 124-31.

21. McArdle PD, Greenfield SM, Rilstone SK, Narendran P, Haque MS, Gill PS. Carbohydrate restriction for glycaemic control in Type 2 diabetes: a systematic review and meta-analysis. *Diabet Med* 2019; **36**(3): 335-48.
22. Korsmo-Haugen HK, Brurberg KG, Mann J, Aas AM. Carbohydrate quantity in the dietary management of type 2 diabetes: A systematic review and meta-analysis. *Diabetes Obes Metab* 2019; **21**(1): 15-27.
23. van Zuuren EJ, Fedorowicz Z, Kuijpers T, Pijl H. Effects of low-carbohydrate- compared with low-fat-diet interventions on metabolic control in people with type 2 diabetes: a systematic review including GRADE assessments. *Am J Clin Nutr* 2018; **108**(2): 300-31.
24. Sainsbury E, Kizirian NV, Partridge SR, Gill T, Colagiuri S, Gibson AA. Effect of dietary carbohydrate restriction on glycemic control in adults with diabetes: A systematic review and meta-analysis. *Diabetes Res Clin Pract* 2018; **139**: 239-52.
25. Naude CE, Schoonees A, Senekal M, Young T, Garner P, Volmink J. Low carbohydrate versus isoenergetic balanced diets for reducing weight and cardiovascular risk: a systematic review and meta-analysis. *PLoS ONE* 2014; **9**(7): e100652.
26. Goldenberg JZ, Day A, Brinkworth GD, et al. Efficacy and safety of low and very low carbohydrate diets for type 2 diabetes remission: systematic review and meta-analysis of published and unpublished randomized trial data. *BMJ* 2021; **372**: m4743.
27. Noronha JC, Nishi SK, Braunstein CR, et al. The Effect of Liquid Meal Replacements on Cardiometabolic Risk Factors in Overweight/Obese Individuals With Type 2 Diabetes: A Systematic Review and Meta-analysis of Randomized Controlled Trials. *Diabetes Care* 2019; **42**(5): 767-76.
28. Rehackova L, Arnott B, Araujo-Soares V, Adamson AA, Taylor R, Sniehotta FF. Efficacy and acceptability of very low energy diets in overweight and obese people with Type 2 diabetes mellitus: a systematic review with meta-analyses. *Diabet Med* 2016; **33**(5): 580-91.
29. Churuangsuk C, Kherouf M, Combet E, Lean M. Low-carbohydrate diets for overweight and obesity: a systematic review of the systematic reviews. *Obes Rev* 2018; **19**(12): 1700-18.
30. Lean ME, Leslie WS, Barnes AC, et al. Primary care-led weight management for remission of type 2 diabetes (DiRECT): an open-label, cluster-randomised trial. *Lancet* 2018; **391**(10120): 541-51.
31. Lean MEJ, Leslie WS, Barnes AC, et al. Durability of a primary care-led weight-management intervention for remission of type 2 diabetes: 2-year results of the DiRECT open-label, cluster-randomised trial. *Lancet Diabetes Endocrinol* 2019; **7**(5): 344-55.
32. Taheri S, Zaghloul H, Chagoury O, et al. Effect of intensive lifestyle intervention on bodyweight and glycaemia in early type 2 diabetes (DIADEM-I): an open-label, parallel-group, randomised controlled trial. *Lancet Diabetes Endocrinol* 2020; **8**(6): 477-89.
33. Esposito K, Maiorino MI, Petrizzo M, Bellastella G, Giugliano D. The effects of a Mediterranean diet on the need for diabetes drugs and remission of newly diagnosed type 2 diabetes: follow-up of a randomized trial. *Diabetes Care* 2014; **37**(7): 1824-30.
34. Gregg EW, Chen H, Wagenknecht LE, et al. Association of an intensive lifestyle intervention with remission of type 2 diabetes. *JAMA* 2012; **308**(23): 2489-96.
35. Gutierrez-Mariscal FM, Cardelo MP, de la Cruz S, et al. Reduction in Circulating Advanced Glycation End Products by Mediterranean Diet Is Associated with Increased Likelihood of Type 2 Diabetes Remission in Patients with Coronary Heart Disease: From the Cordioprev Study. *Mol Nutr Food Res* 2021; **65**(1): e1901290.
36. Hallberg SJ, McKenzie AL, Williams PT, et al. Effectiveness and Safety of a Novel Care Model for the Management of Type 2 Diabetes at 1 Year: An Open-Label, Non-Randomized, Controlled Study. *Diabetes Ther* 2018; **9**(2): 583-612.
37. Bynoe K, Unwin N, Taylor C, et al. Inducing remission of Type 2 diabetes in the Caribbean: findings from a mixed methods feasibility study of a low-calorie liquid diet-based intervention in Barbados. *Diabet Med* 2020; **37**(11): 1816-24.
38. Dave RD, R.;Davies, J. S. The impact of multiple lifestyle interventions on remission of type 2 diabetes mellitus within a clinical setting. *Obes Med* 2019; **13**: 59-64.

39. Mottalib A, Sakr M, Shehabeldin M, Hamdy O. Diabetes Remission after Nonsurgical Intensive Lifestyle Intervention in Obese Patients with Type 2 Diabetes. *J Diabetes Res* 2015; **2015**: 468704.
40. Sarathi V, Kolly A, Chaithanya HB, Dwarakanath CS. High rates of diabetes reversal in newly diagnosed Asian Indian young adults with type 2 diabetes mellitus with intensive lifestyle therapy. *J Nat Sci Biol Med* 2017; **8**(1): 60-3.
41. Steven S, Hollingsworth KG, Al-Mrabeh A, et al. Very Low-Calorie Diet and 6 Months of Weight Stability in Type 2 Diabetes: Pathophysiological Changes in Responders and Nonresponders. *Diabetes Care* 2016; **39**(5): 808-15.
42. Thomas ND, Shamanna SB. Impact of calorie restriction on glycemic control in overweight patients with type 2 diabetes mellitus. *Int J Diabetes Dev C* 2018; **39**(3): 547-50.
43. Umphonsathien M, Prutanopajai P, Aiam ORJ, et al. Immediate and long-term effects of a very-low-calorie diet on diabetes remission and glycemic control in obese Thai patients with type 2 diabetes mellitus. *Food Sci Nutr* 2019; **7**(3): 1113-22.
44. Vitale R, Kim Y. The Effects of Intermittent Fasting on Glycemic Control and Body Composition in Adults with Obesity and Type 2 Diabetes: A Systematic Review. *Metab Syndr Relat Disord* 2020; **18**(10): 450-61.
45. Welton S, Minty R, O'Driscoll T, et al. Intermittent fasting and weight loss: Systematic review. *Can Fam Physician* 2020; **66**(2): 117-25.
46. Balshem H, Helfand M, Schunemann HJ, et al. GRADE guidelines: 3. Rating the quality of evidence. *J Clin Epidemiol* 2011; **64**(4): 401-6.
47. Carter S, Clifton PM, Keogh JB. The effects of intermittent compared to continuous energy restriction on glycaemic control in type 2 diabetes; a pragmatic pilot trial. *Diabetes Res Clin Pract* 2016; **122**: 106-12.
48. Carter S, Clifton PM, Keogh JB. Effect of Intermittent Compared With Continuous Energy Restricted Diet on Glycemic Control in Patients With Type 2 Diabetes: A Randomized Noninferiority Trial. *JAMA Netw Open* 2018; **1**(3): e180756.
49. Kahleova H, Belinova L, Malinska H, et al. Eating two larger meals a day (breakfast and lunch) is more effective than six smaller meals in a reduced-energy regimen for patients with type 2 diabetes: a randomised crossover study. *Diabetologia* 2014; **57**(8): 1552-60.
50. Delgado-Lista J, Perez-Martinez P, Garcia-Rios A, et al. CORonary Diet Intervention with Olive oil and cardiovascular PREVention study (the CORDIOPREV study): Rationale, methods, and baseline characteristics: A clinical trial comparing the efficacy of a Mediterranean diet rich in olive oil versus a low-fat diet on cardiovascular disease in coronary patients. *Am Heart J* 2016; **177**: 42-50.
51. Mollentze WF, Joubert G, Prins A, van der Linde S, Marx GM, Tsie KG. The safety and efficacy of a low-energy diet to induce weight loss, improve metabolic health, and induce diabetes remission in insulin-treated obese men with type 2 diabetes: a pilot RCT. *Int J Diabetes Dev C* 2019; **39**(4): 618-25.
52. Athinarayanan SJ, Adams RN, Hallberg SJ, et al. Long-Term Effects of a Novel Continuous Remote Care Intervention Including Nutritional Ketosis for the Management of Type 2 Diabetes: A 2-Year Non-randomized Clinical Trial. *Front Endocrinol (Lausanne)* 2019; **10**: 348.
